# An Event-Based Solution to the Perspective-*n*-Point Problem

**DOI:** 10.3389/fnins.2016.00208

**Published:** 2016-05-18

**Authors:** David Reverter Valeiras, Sihem Kime, Sio-Hoi Ieng, Ryad Benjamin Benosman

**Affiliations:** Centre National de la Recherche Scientifique, Institut National de la Santé et de la Recherche Médicale, Institut de la Vision, Sorbonne Universités, UPMC Université Paris 06Paris, France

**Keywords:** neuromorphic vision, event-based imaging, 3D pose estimation, P*n*P problem, visual tracking

## Abstract

The goal of the Perspective-*n*-Point problem (P*n*P) is to find the relative pose between an object and a camera from a set of *n* pairings between 3D points and their corresponding 2D projections on the focal plane. Current state of the art solutions, designed to operate on images, rely on computationally expensive minimization techniques. For the first time, this work introduces an event-based P*n*P algorithm designed to work on the output of a neuromorphic event-based vision sensor. The problem is formulated here as a least-squares minimization problem, where the error function is updated with every incoming event. The optimal translation is then computed in closed form, while the desired rotation is given by the evolution of a virtual mechanical system whose energy is proven to be equal to the error function. This allows for a simple yet robust solution of the problem, showing how event-based vision can simplify computer vision tasks. The approach takes full advantage of the high temporal resolution of the sensor, as the estimated pose is incrementally updated with every incoming event. Two approaches are proposed: the Full and the Efficient methods. These two methods are compared against a state of the art P*n*P algorithm both on synthetic and on real data, producing similar accuracy in addition of being faster.

## 1. Introduction

The Perspective-*n*-Point problem—usually referred to as P*n*P—is the problem of finding the relative pose between an object and a camera from a set of *n* pairings between 3D points of the object and their corresponding 2D projections on the focal plane, assuming that a model of the object is available. Since it was formally introduced in 1981 (Fischler and Bolles, 1981), the P*n*P problem has found numerous applications in photogrammetry and computer vision, such as tracking (Lepetit and Fua, 2006), visual servoing (Montenegro Campos and de Souza Coelho, 1999), or augmented reality (Skrypnyk and Lowe, 2004).

For three or four points with non-collinear projections on the focal plane, the P*n*P problem can be solved up to some ambiguity in the camera pose (Haralick et al., 1994). When more points are to be considered, the standard method is to minimize the sum of some squared error, usually defined on the focal plane. Existing methods differ in the way this error function is minimized, and can be classified as iterative (see Dementhon and Davis, 1995) and non-iterative (Lepetit et al., 2008). Other techniques consider an object-space error function instead (Lu et al., 2000; Schweighofer and Pinz, 2006). The advantage of such an approach is that a correct matching of the 3D points leads to a correct 2D projection on the focal plane, while the reverse is not necessarily true.

Current methods, designed to work on images, are inevitably limited by the low frame rates of conventional cameras, usually in the range of 30–60 Hz. The frame based stroboscopic acquisition induces redundant data and temporal gaps that make it difficult to estimate the pose of a 3D object without computationally expensive iterative optimization techniques (Chong and Zak, 2001). Frame-based methods are suitable for many applications, as long as the frame rate is able to capture the motion. However, even if the frame rate is sufficient, it is mandatory to process non-relevant information. This paper introduces a new approach designed to work on the output of an asynchronous event-based neuromorphic camera. Neuromorphic cameras are a novel type of vision sensors that operate on a new acquisition paradigm: instead of capturing static images of the scene, they record pixel intensity changes at the precise instants they occur. This results in a high temporal precision that provides information about scene dynamics which introduce a paradigm shift in visual processing, as shown in previous contributions (Clady et al., 2014; Lagorce et al., 2014).

To our knowledge, this is the first P*n*P algorithm designed to work on the asynchronous output of neuromorphic event-based vision sensors. In Ni et al. (2014), an event-based iterative closest point (ICP) like tracking algorithm is introduced, where the pattern is a 2D point cloud which is updated with every incoming event, so that it matches the projection of a given object. However, their solution assumes that the pattern is undergoing some affine transformation (Coxeter, 1961), and therefore it does not account for a more general transformation due to perspective projection that an object freely evolving in the 3D space can experiment. Furthermore, the pose of the object in the 3D space is never estimated. The work of Ni et al. was extended in Reverter Valeiras et al. (2016), where we presented an event-based 3D pose estimation algorithm. In Reverter Valeiras et al. (2016), the model of an object is given as a collection of points, edges and faces, and iteratively attracted toward the line of sight of every incoming event. However, the method is based on the assumption that the estimation is always close to the true pose of the object, and thus requires a manual initialization step. The technique described in the present paper, greatly inspired by the work of Lu et al. (2000), is designed to overcome this limitation.

The main motivation of this work is its application to visual servoing. Our goal is to build a perception-action loop fast and efficient enough to match the performance of biological sensory-motor systems. From past experiences, neuromorphic sensing techniques seem to be the most promising way to reach this goal.

## 2. Materials and methods

### 2.1. Event-based imaging

Neuromorphic cameras are a new type of biomimetic vision sensors, often referred to as “silicon retinas.” Unlike conventional imagers, these devices are not controlled by artificially created clock signals that have no relation to the source of the visual information (Lichtsteiner et al., 2008). Instead, neuromorphic cameras are driven by “events” happening within the scene, and they transmit information in an asynchronous manner, just like the biological eye does.

One of the first attempts of designing a neuromorphic vision sensor incorporating the functionalities of the retina is the pioneering work of Mahowald (1992) in the late eighties. Since then, a variety of these event-based devices have been created, including gradient-based sensors sensitive to static edges (Delbrück, 1993), temporal contrast vision sensors sensitive to relative illuminance change (Lichtsteiner et al., 2008; Posch et al., 2008, 2011), edge-orientation sensitive devices and optical-flow sensors (Etienne-Cummings et al., 1997; Krammer and Koch, 1997). Most of these sensors output compressed digital data in the form of asynchronous address events (AER; Boahen, 2000), removing redundancy, reducing latency and increasing dynamic range as compared with traditional imagers. A comprehensive review of neuromorphic vision sensors can be found in Delbrück et al. (2010) and Posch et al. (2014).

**Figure 1:**
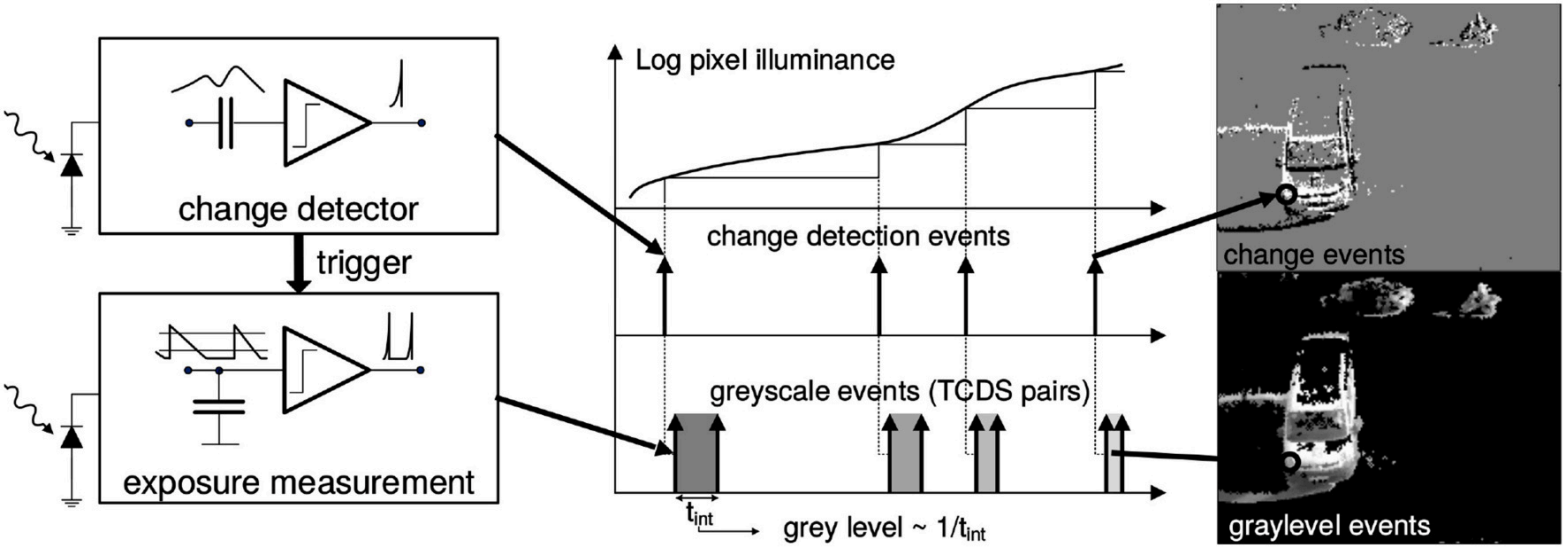
**Functional diagram of an ATIS pixel (Posch et al., 2011)**. Two types of asynchronous events, encoding change (top) and illuminance (bottom) information, are generated and transmitted individually by each pixel in the imaging array when a change is detected in the scene. The bottom right image only shows grayscale of pixels for which illuminance has recently been measured. Black pixels indicate locations where illuminance has not been measured recently.

The presented solution to the P*n*P problem is designed to work with such sensors, and it takes full advantage of the sparse data representation and high temporal resolution of their output. The Asynchronous Time Image Sensor (ATIS) used in this work is a time-domain encoding vision sensor with 304 × 240 pixels resolution (Posch et al., 2008). The sensor contains an array of fully autonomous pixels that combine an illuminance change detector circuit and a conditional exposure measurement block. As shown in the functional diagram of the ATIS pixel in Figure 1, the change detector individually and asynchronously initiates the measurement of an exposure/gray scale value only if—and immediately after—an illuminance change of a certain magnitude has been detected in the field-of-view of the respective pixel. The exposure measurement circuit in each pixel individually encodes the absolute pixel illuminance into the timing of asynchronous event pulses, more precisely into inter-event intervals.

Because the ATIS reads out events as they happen, its temporal resolution is highly accurate—on the order of microseconds. The time-domain encoding of the intensity information automatically optimizes the exposure time separately for each pixel instead of imposing a fixed integration time for the entire array, resulting in an exceptionally high dynamic range and improved signal to noise ratio. The individual pixel change detector operation yields almost ideal temporal redundancy suppression, resulting in a sparse encoding of the image data. Frames are absent from this acquisition process. They can however be reconstructed, when needed, at frequencies limited only by the temporal resolution of the pixel circuits (up to hundreds of kiloframes per second). Reconstructed images from the sensor have been used for display purposes.

The stream of events can be mathematically described as follows: let $e_{k}={\left(u_{k}^{T},t_{k},p_{k}\right)}^{T}$ be a quadruplet describing an event occurring at time *t*_*k*_ at the position $u_{k}={\left(x_{k},y_{k}\right)}^{T}$ on the focal plane. The two possible values for *p*_*k*_ are 1 or −1, depending on whether a positive or negative change of illuminance has been detected.

### 2.2. Event-based solution to the P*n*P problem

#### 2.2.1. Problem formulation

**Figure 2:**
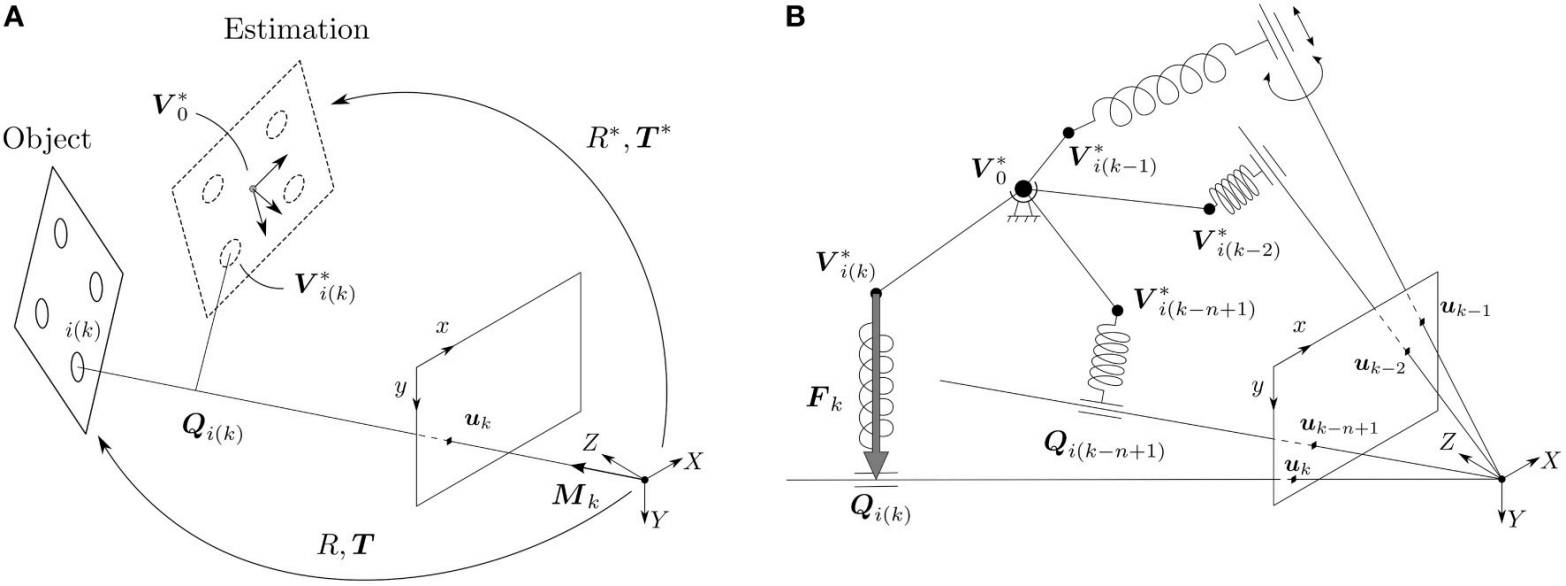
**(A)** An object, given as a collection of 3D points {***V***_*i*_}, is observed by a calibrated silicon retina. The true pose of the object is given by (*R*, ***T***), while the estimated pose is denoted (R^*^, T^*^). Attached to the estimation there is a frame of reference, whose origin we denote by ***V***_0_*. An event *e*_*k*_ has to be generated by a point lying on the *line of sight* of the event, whose direction is given by the vector ***M***_*k*_. The point of the object generating event *e*_*k*_ is denoted by the index *i*(*k*). ***Q***_*i*(*k*)_ is then the projection of V^*^_i(*k*)_ on its corresponding line of sight. When the estimation is aligned with the true position of the object, then ***Q***_*i*(*k*)_ and V^*^_i(*k*)_ are the same. **(B)** In order to solve the rotation, we build the following virtual mechanical system: the origin of the estimation ***V***_0_* is linked to the world by a spherical joint, and every point of the estimation V^*^_i(*k*−*j*)_ generating an event *e*_*k*−*j*_ is linked to its corresponding line of sight by a linear spring. Simulating the behavior of this mechanical system is equivalent to minimizing the collinearity error.

Let us imagine a scene with a moving rigid object observed from a calibrated silicon retina, as shown in Figure 2A. Let {***V***_*i*_} be a model of the object, described as a collection of 3D points $V_{i}={\left(X_{i},Y_{i},Z_{i}\right)}^{T}$. Attached to this object there is a frame of reference, whose origin we denote as ***V***_0_.

The pinhole projection maps 3D points ***V***_*i*_ expressed in the object's frame of reference into ***v***_*i*_ on the camera's focal plane, according to the relation:

(1) $$\left(\begin{array}{c} v_{i}\\ 1 \end{array}\right)\sim K(R\;\;T)\left(\begin{array}{c} V_{i}\\ 1 \end{array}\right),$$

where *K* is the 3 × 3 matrix defining the camera's intrinsic parameters—obtained through a prior calibration procedure —and *R* ∈ *SO*(3), **T** ∈ ℝ^3^ are the extrinsic ones. The sign ~ indicates that the equality is defined up to a scale (Hartley and Zisserman, 2003). (*R*, **T**) are also referred to as the relative pose between the object and the camera (Murray et al., 1994). As the object moves, only the pose changes and needs to be estimated. We will denote (R^*^, T^*^) our current estimation of the pose, that we update with the incoming events. The corresponding points of the estimation are denoted as $V_{i}^{*}$ and computed with the expression:

(2) $$\begin{array}{l} V_{i}^{*}=R^{*}V_{i}+T^{*}. \end{array}$$

Analogously, the origin of the frame of reference attached to the estimation is denoted as $V_{0}^{*}$.

#### 2.2.2. Rotation formalisms

Rotation matrices are one of the most widely used formalisms for representing rotations, and they will be employed throughout the whole article for developing our algorithm. However, some other representations are possible, each one with its own advances and disadvantages (Murray et al., 1994). For example, rotations matrices are not well suited for visualization. Hence, when trying to visualize rotations we will use the axis-angle representation, where the rotation is expressed as the rotation vector $r=\phi \tilde{\text{r}}$, where $\tilde{r}$ is a unit vector in the direction of the axis of rotation and ϕ is the rotation angle.

#### 2.2.3. Object-space collinearity error

Let us consider an event $e_{k}={\left(u_{k}^{T},t_{k},p_{k}\right)}^{T}$ occurring at time *t*_*k*_ at location $u_{k}={\left(x_{k},y_{k}\right)}^{T}$. According to the pinhole camera model, we know that this event has to be generated by a point lying on the *line of sight* of event *e*_*k*_—the line defined by the optical center and the spatial position of the event on the focal plane—as shown in Figure 2A. Assuming that we can identify which point of the object has generated the event, we try to estimate the pose that minimizes the orthogonal projection errors on the line of sight for the last *n* events.

Let ***M***_*k*_ be a vector defining the line of sight of event *e*_*k*_, whose coordinates can be easily obtained as:

(3) $$M_{k}=K^{-1}\left(\begin{array}{c} u_{k}\\ 1 \end{array}\right).$$

Next, let us assume that we can identify the point of the object that has generated event *e*_*k*_, that we denote by the index *i*(*k*). Hence, if the true pose of the object and the estimation were perfectly aligned, $V_{i\left(k\right)}^{*}$ would necessarily lie on the line of sight of event *e*_*k*_.

Let ***Q***_*i*(*k*)_ be the projection of $V_{i\left(k\right)}^{*}$ on the line of sight of event *e*_*k*_, that can be computed as:

(4) $$Q_{i(k)}=\frac{{\left(V_{i(k)}^{*}\right)}^{T}M_{k}}{\|M_{k}{\|}^{2}}M_{k}=L_{k}V_{i\;(k)}^{*},$$

where *L*_*k*_ is the *Line-of-Sight* projection matrix of event *e*_*k*_ that takes the value:

(5) $$\begin{array}{l} L_{k}=\frac{M_{k}M_{k}^{T}}{M_{k}^{T}M_{k}}. \end{array}$$

For a given event *e*_*k*_, we define the object-space collinearity error **ξ**_*k*_(R^*^, T^*^) as:

(6) $$\begin{array}{l} \xi_{k}\left(R^{*},T^{*}\right)=Q_{i\left(k\right)}-V_{i\left(k\right)}^{*}=\left(L_{k}-I_{3}\right)V_{i\left(k\right)}^{*}, \end{array}$$

where *I*_3_ denotes the 3 × 3 identity matrix. We will take into account the last *n* events and minimize the sum of the squared collinearity errors, where errors can be weighted. The goal of our algorithm will therefore be to minimize the following error function $E_{k}\left(R^{*},T^{*}\right)$:

(7) $$\begin{array}{l} E_{k}\left(R^{*},T^{*}\right)=\frac{1}{W}\overset{n-1}{\underset{j=0}{\sum }}w_{j}||\xi_{k-j}\left(R^{*},T^{*}\right)|{|}^{2}, \end{array}$$

where $\xi_{k-j}\left(R^{*},T^{*}\right)$ denotes the collinearity error of event *e*_*k*−*j*_ (that is to say, the event occurring *j* steps before the current one) with *j* = 0, 1, …, *n* − 1. The weight of the corresponding error is then denoted *w*_*j*_, verifying:

(8a) $$\begin{array}{r} w_{j}\geq 0,\;\forall j=0,1,\ldots ,n-1, \end{array}$$

(8b) $$\begin{array}{r} W=\overset{n-1}{\underset{j=0}{\sum }}w_{j}. \end{array}$$

In general, we will set the weights *w*_*j*_ so that they decrease with *j*, giving a greater importance to the most recent events. Here, *W* is just a normalizing factor, whose value is usually imposed to be 1.

We will therefore be looking for the pose (R^*^, T^*^) that minimizes the error function given by Equation (7), which is computed as the sum of the collinearity errors for the last *n* events. These collinearity errors depend on the estimated position of the point generating the event (where we assume that we can identify the point) and the position of the corresponding event on the focal plane (or, equivalently, the projection of the point generating the event). Consequently, our approach can be classified as a solution to the P*n*P problem, since we are estimating the pose of an object from a set of *n* pairings between 3D points of the object and their projections on the focal plane. Unlike classical frame-based techniques, our approach allows us to consider several events generated by the same point of the object, and thus *n* can be chosen to be bigger than the number of points conforming the object.

**Remark:** P*n*P methods always require matching 3D points with their corresponding 2D projections. In the case of our method, this equals to identifying which point of the object has generated an event. Consequently, our algorithm relies on an event-based tracking technique. For the rest of this paper, the term “tracking” will always refer to this previous method. As we will show in the experiments, the overall performance of the system is strongly dependent on the accuracy of this tracking.

#### 2.2.4. Translation

For a given rotation R^*^, the optimal translation that minimizes the sum of the squared collinearity errors can be computed in closed form Lu et al. (2000). Equivalently, for a given estimation (R^*^, T^*^), the optimal displacement Δ***T***_*k*_ can be computed as:

(9) $$\begin{array}{l} \Delta T_{k}\left(R^{*},T^{*}\right)=A_{k}^{-1}B_{k}, \end{array}$$

where *A*_*k*_ is a 3 × 3 matrix and ***B***_*k*_ a 3D vector given by:

(10) $$\begin{array}{r} A_{k}=\overset{n-1}{\underset{j=0}{\sum }}w_{j}\left(I_{3}-L_{k-j}\right), \end{array}$$

(11) $$\begin{array}{r} B_{k}=\overset{n-1}{\underset{j=0}{\sum }}w_{j}\left(L_{k-j}-I_{3}\right)V_{i\left(k-j\right)}^{*}. \end{array}$$

We will refer to this way of computing *A*_*k*_ and ***B***_*k*_ as the *full method*. As shown in Lu et al. (2000), *A*_*k*_ can be proven to be non-singular, guaranteeing that Equation (9) can always be solved. We then update the estimation of the position making:

(12) $$\begin{array}{l} T_{k}^{*}=T_{k-1}^{*}+\lambda_{T}\Delta T_{k}, \end{array}$$

where $T_{k}^{*}$ denotes the estimated translation at time *t*_*k*_, and λ_*T*_ is a tuning factor. Let us note that λ_*T*_ is a dimensionless quantity, and it should always be chosen smaller or equal to one. Its effect will be more carefully studied in the experiments.

As shown in the Appendix A in Supplementary Material, for a correctly chosen set of weights and under some reasonable assumptions, *A*_*k*_ and ***B***_*k*_ can be iteratively updated making:

(13) $$\begin{array}{r} A_{k}\approx w_{0}\left(I_{3}-L_{k}\right)+\left(1-w_{0}\right)A_{k-1}, \end{array}$$

(14) $$\begin{array}{r} B_{k}\approx w_{0}\left(L_{k}-I_{3}\right)V_{i\left(k\right)}^{*}+\left(1-w_{0}\right)B_{k-1}. \end{array}$$

This allows us to update *A*_*k*_ and ***B***_*k*_ for each event in an iterative manner, saving memory and computation time. We will refer to this way of updating *A*_*k*_ and ***B***_*k*_ as the *efficient method*, and test its effect on the experiments.

#### 2.2.5. Rotation

As shown in Lu et al. (2000), for a given translation T^*^ the optimal rotation R^*^ cannot be computed in closed form. In Lu et al. (2000), the rotation is obtained via an absolute orientation problem between the points of the estimation and their projections onto the corresponding line of sight, which is then solved using Singular Value Decomposition. This is, however, a computationally expensive process, not well-suited to the output of the neuromorphic camera: in order to fully exploit the high dynamics of the sensor, we wish to update the estimated pose with every incoming event. Given the high frequency of the arrival of events, the computations carried out with each one of them should be kept to a minimum, in order to achieve real-time performance.

In our approach, instead of trying to find the optimal rotation for each event, we will simply apply a rotation such that our error function is reduced at each step. Since events happen with such a high temporal resolution, this will very fast lead to a correct estimation. To that end, we will define a virtual mechanical system whose energy is equal to the error function. Since mechanical systems evolve in the sense of minimizing their energy, simulating the behavior of this system will be equivalent to minimizing the error function, approaching the estimation toward its true value.

Consequently, let us picture the following virtual mechanical system: since rotations happen around the origin of the estimation $V_{0}^{*}$, let us imagine $V_{0}^{*}$ to be attached to the world by a spherical joint, as shown in Figure 2B. This allows the object to freely rotate around this point, but prevents any translation. Next, for every event *e*_*k*−*j*_ with *j* = 0, 1, …, *n* − 1 (that is to say, for the last *n* events) we wish to attract the corresponding point of the estimation $V_{i\left(k-j\right)}^{*}$ toward the line of sight of the event. To that end, let us imagine $V_{i\left(k-j\right)}^{*}$ and the line of sight to be linked by a linear spring, whose direction is always perpendicular to the line of sight. In other words, we link $V_{i\left(k-j\right)}^{*}$ and ***Q***_*i*(*k*−*j*)_ by a linear spring. In a real mechanical system, this would be achieved by linking the spring and the line of sight with a cylindrical joint, as shown in Figure 2B.

The force ***F***_*k*−*j*_ exerted by a linear spring is given by Hooke's law, which states that the direction of the force is that of the axis of the spring, and its magnitude is given by the expression:

(15) $$\begin{array}{l} ||F_{k-j}||=C_{k-j}\Delta l_{k-j}=C_{k-j}\left(l_{k-j}-l_{0}\right), \end{array}$$

where *C*_*k*−*j*_ is the stiffness of the spring and Δ*l*_*k*−*j*_ = *l*_*k*−*j*_ − *l*_0_ its elongation. Since the axis of the spring is aligned with $Q_{i\left(k-j\right)}-V_{i\left(k-j\right)}^{*}$, and considering Equation (6), ***F***_*k*−*j*_ takes the value:

(16) $$\begin{array}{l} F_{k-j}=C_{k-j}\frac{\xi_{k-j}}{\left||\xi_{k-j}|\right|}\Delta l_{k-j}. \end{array}$$

Next, let us make *l*_0_ = 0. This implies that the elongation at rest is zero. In other words, the virtual spring will not produce any force when $V_{i\left(k-j\right)}^{*}$ lies on its corresponding line of sight, that is to say when it is correctly aligned with the corresponding event. The elongation Δ*l*_*k*−*j*_ then takes the value:

(17) $$\begin{array}{l} \Delta l_{k-j}=l_{k-j}=||Q_{i\left(k-j\right)}-V_{i\left(k-j\right)}^{*}||=||\xi_{k-j}||. \end{array}$$

Finally, let us make the magnitude of the stiffness equal to the weight of the corresponding event:

(18) $$\begin{array}{l} C_{k-j}=\beta w_{j}, \end{array}$$

where β is just a unit adjustment constant, that compensates for the fact that weights are dimensionless, but not the stiffness. For the rest of this paper all distances will be given in mm, and thus β = 1 Nmm^-1^. ***F***_*k*−*j*_ becomes:

(19) $$\begin{array}{l} F_{k-j}=\beta w_{j}\xi_{k-j}. \end{array}$$

Let us remind the reader that the energy *g*_*k*−*j*_ of a linear spring is given by the expression:

(20) $$\begin{array}{l} g_{k-j}=\frac{1}{2}C_{k-j}{\left(\Delta l_{k-j}\right)}^{2}. \end{array}$$

The energy *G*_*k*_ of the whole system, when considering the last *n* events, is computed by applying the principle of superposition:

(21) $$\begin{array}{l} G_{k}=\overset{n-1}{\underset{j=0}{\sum }}g_{k-j}=\frac{1}{2}\overset{n-1}{\underset{j=0}{\sum }}\beta w_{j}||\xi_{k-j}|{|}^{2}=\frac{\beta W}{2}E_{k}, \end{array}$$

whose magnitude is equal to the error function, up to some normalization factor. Simulating the behavior of this system will then be equivalent to minimizing the error function.

Since the translation is prevented in this case, we only wish to compute the moments of the forces and their effect. Thus, let **τ**_*k*−*j*_ be the torque generated by force ***F***_*k*−*j*_ with respect to the origin of the estimation $V_{0}^{*}$:

(22) $$\begin{array}{l} \begin{array}{c} \tau_{k-j}=\left(V_{i\left(k-j\right)}^{*}-V_{0}^{*}\right)\times F_{k-j}\\ =R^{*}V_{i\left(k-j\right)}\times \beta w_{j}\left(L_{k-j}-I_{3}\right)V_{i\left(k-j\right)}^{*}, \end{array} \end{array}$$

where × denotes the cross product. The resulting torque **Γ**_*k*_ when we take into account the last *n* events takes the value:

(23) $$\begin{array}{l} \Gamma_{k}=\overset{n-1}{\underset{j=0}{\sum }}R^{*}V_{i\left(k-j\right)}\times \beta w_{j}\left(L_{k-j}-I_{3}\right)V_{i\left(k-j\right)}^{*}. \end{array}$$

We will compute the resulting torque using this expression when applying the *full method*. We then approximate its effect by a rotation given, in its axis-angle representation, by the vector ***r***_*k*_ computed as:

(24) $$\begin{array}{l} r_{k}=\lambda_{r}\Gamma_{k}, \end{array}$$

where λ_*r*_ is a tuning factor. A complete justification of this choice is given in the Appendix B in Supplementary Material.

In the Appendix C in Supplementary Material we give some more insight on how to pick a value for λ_*r*_, and derive the following expression for its theoretical optimum $\lambda_{r}^{opt}$:

(25) $$\begin{array}{l} \lambda_{r}^{opt}=\frac{3\pi }{2\left(1+\sqrt{2}\right)}\frac{1}{\beta W\rho_{max}^{2}}, \end{array}$$

where ρ_*max*_ is equal to the maximum distance in the object $\rho_{max}=\underset{i}{\max}\{\|V_{i}\|\}$. From this expression it is evident that λ_*r*_ is not dimensionless, and its optimal value will therefore depend on the dimensions of the object whose pose we want to estimate (and the units in which they are expressed). For the rest of this paper, all values of λ_*r*_ will be expressed in N^-1^mm^-1^.

Let Δ*R*_*k*_ be the rotation matrix corresponding to the rotation represented by ***r***_*k*_. We update the estimation with the following expression:

(26) $$\begin{array}{l} R_{k}^{*}=\Delta R_{k}R_{k-1}^{*}. \end{array}$$

As in the case of *A*_*k*_ and ***B***_*k*_, the resulting torque can be approximated with the iterative expression:

(27) $$\begin{array}{l} \Gamma_{k}\approx \beta w_{0}R^{*}V_{i\left(k\right)}\times \left(L_{k}-I_{3}\right)V_{i\left(k\right)}^{*}+\left(1-w0\right)\Gamma_{k-1}. \end{array}$$

The value of the resulting torque will be updated in this way when applying the *efficient method*.

#### 2.2.6. Global algorithm

The P*n*P problem is solved by the global algorithm described below.

## 3. Results

In this section, two experiments showing the accuracy of our method are presented. The algorithm is implemented in Matlab and C++ and tested in a synthetic scene for the first experiment. Next, another experiment is produced from a real recording.

In order to characterize the accuracy of our method we will consider the sum of the squared collinearity errors *E*_*k*_. Additionally, we adopt the following metrics in the space of rigid motions:

- The absolute estimation error in linear translation is given by the norm of the difference between the estimated translation T^*^ and its true value ***T***. We define the relative translation error ξ_*T*_ as:
  (28) $$\begin{array}{l} \xi_{T}\left(\%\right)=100\;\frac{\left||T^{*}-T|\right|}{\left||\bar{T}|\right|}, \end{array}$$
  where $\left||\bar{T}|\right|$ is the norm of the mean translation of the object for the whole experiment.
- The distance *d* between two rotations, given by the corresponding rotation matrices *R*_1_ and *R*_2_ can be computed as:
  (29) $$\begin{array}{l} d\left(R_{1},R_{2}\right)=||I_{3}-R_{1}R_{2}^{T}|{|}_{F}, \end{array}$$
  where *I*_3_ is the 3 × 3 identity matrix and || · ||_*F*_ denotes the Frobenius norm of the matrix. This can be proven to be a metric in the space of 3D rotations (Huynh, 2009) and takes values in the range $\left[0,2\sqrt{2}\right]$. Thus, let ξ_*R*_ be the relative rotation error computed as:
  (30) $$\begin{array}{l} \xi_{R}\left(\%\right)=100\;\frac{d\left(R^{*},R\right)}{2\sqrt{2}}. \end{array}$$

**Algorithm 1:** Event-Based P*n*P algorithm

**Require**: $e_{k}={\left(u_{k}^{T},t_{k},p_{k}\right)}^{T}$∀*k* > 0
**Ensure**: R^*^, T^*^
Initialize the parameters
**for** every incoming event *e_k_* **do**
Identify the point generating the event *i(k)*
Compute the *Line-of-Sight* projection matrix *L_k_* using Equation (5)
**if** *full* *method* **then**
Compute the resulting torque **Γ**_*k*_ using Equation (23)
Compute *A_k_* and ***B**_k_* using Equations (10) and (11)
**else if** *efficient* *method* **then**
Update the resulting torque **Γ**_*k*_ using Equation (27)
Update *A_k_* and ***B**_k_* using Equations (13) and (14)
**end if**
Compute the resulting rotation Δ*R_k_* using Equation (24)
Compute the resulting displacement Δ***T**_k_* using Equation (9)

Update R^*^ using Equation (26)
Update T^*^ using Equation (12)
**end for**


For all of the following experiments the weights of the past events are chosen to be linearly decaying. Imposing *W* = 1 yields:

(31) $$\begin{array}{l} w_{j}=\frac{2\left(n-j\right)}{n\left(n+1\right)},\;\forall j=0,\ldots ,n-1. \end{array}$$

### 3.1. Synthetic scene

**Figure 3:**
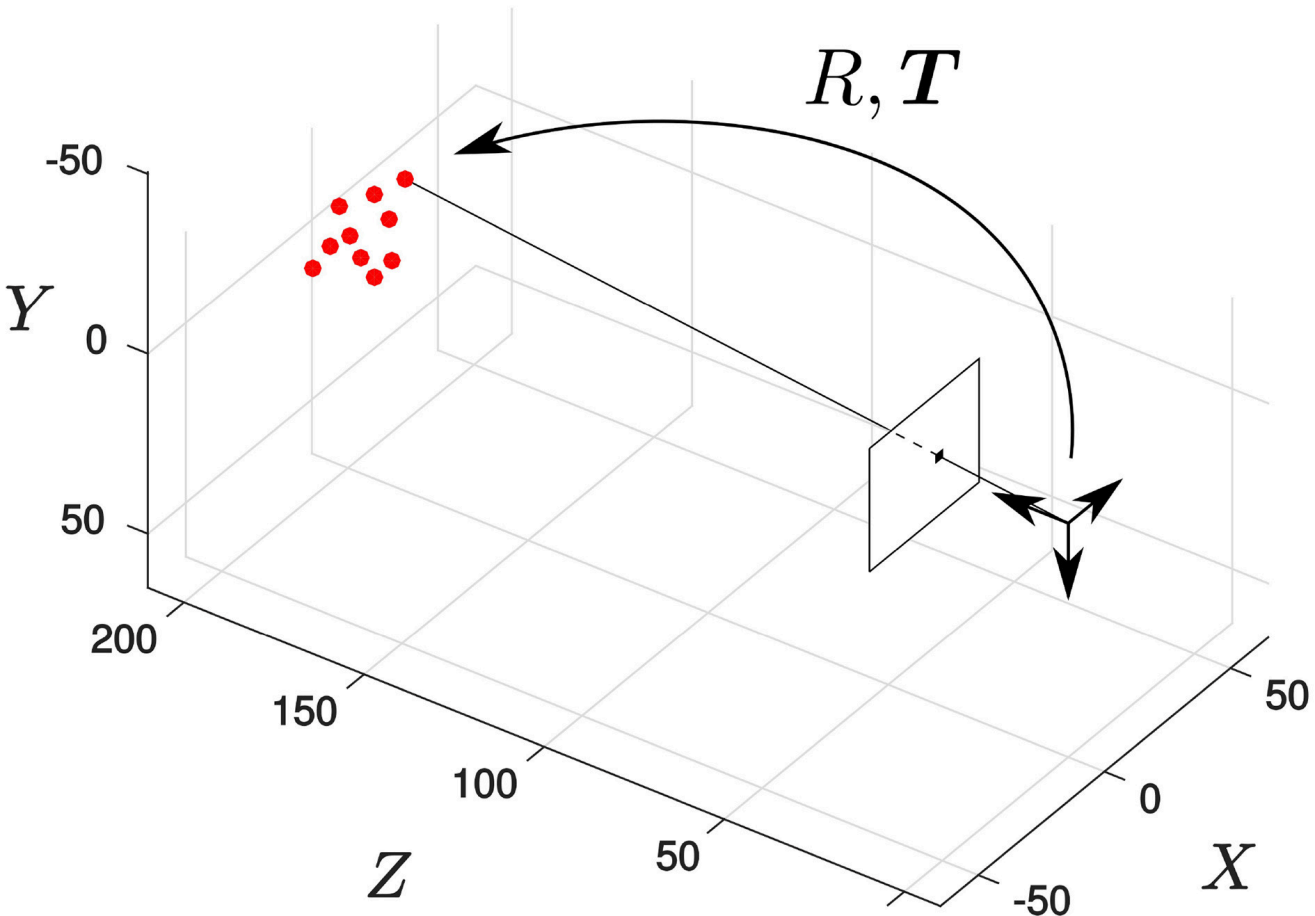
**Synthetic scene: the solid dots represent the pose of the object, static in this experiment**. We randomly select a point of the object and generate an event located on its projection on the focal plane.

The algorithm is first tested in a synthetic scene containing a virtual object. This object is composed by 10 points whose 3D coordinates were randomly initialized following a normal distribution with zero mean and standard deviation equal to 10 mm. Both the object and the camera are assumed to be static, and the pose of the object relative to the camera is given by the translation vector ***T*** = (0, 0, 200)^*T*^ (in mm) and the rotation vector ***r*** = (2∕3, 2∕3, 1∕3)^*T*^. Figure 3 shows the resulting geometry.

The virtual camera has the following intrinsic parameters matrix:

$$K=\left(\begin{array}{ccc} fm_{x} & 0 & c_{x}\\ 0 & fm_{y} & c_{y}\\ 0 & 0 & 1 \end{array}\right),\text{ with }\{\begin{array}{l} f=20\text{ mm}\\ m_{x}=m_{y}=30\text{ px/mm}\\ c_{x}=152\text{ px}\\ c_{y}=120\text{ px} \end{array}$$

which corresponds to an ideal pinhole camera model. The precise geometric parameters are those of an ATIS device equipped with an objective with focal distance 20 mm.

A stream of events is generated from this synthetic scene, sequentially selecting a random point of the object and generating an event on its corresponding projection on the focal plane. The inter-event times are random integers following a normal distribution with mean 5 μs and standard deviation 2 μs (corresponding to some characteristic values observed in ATIS recordings of real moving objects). Let us note, however, that we are not trying to accurately simulate the event generation mechanism of neuromorphic image sensors. For this first experiment we are just trying to evaluate the algorithm when the object is static and assuming perfect tracking. Even if the events are not generated in a realistic fashion, this will allow us to characterize different aspects of the algorithm in the simplest possible situation. We will evaluate our method on real recordings in the next experiment.

In a first step we test the accuracy of the rotation and the translation estimation strategies separately. This will allow us to explore the space of parameters and give some guidance on how to set them.

#### 3.1.1. Translation

In order to exclusively check how the algorithm estimates translation, we make the initial estimation of the rotation R^*^(0) equal to its true value *R*. Additionally, we set the tuning factor for the rotation λ_*r*_ as being zero. Let us remind the reader that the object is in this case static, and thus the estimated rotation will remain equal to its true value at every instant. We make the initial estimation of the translation T^*^(0) = (0, 0, 0)^*T*^.

**Figure 4:**
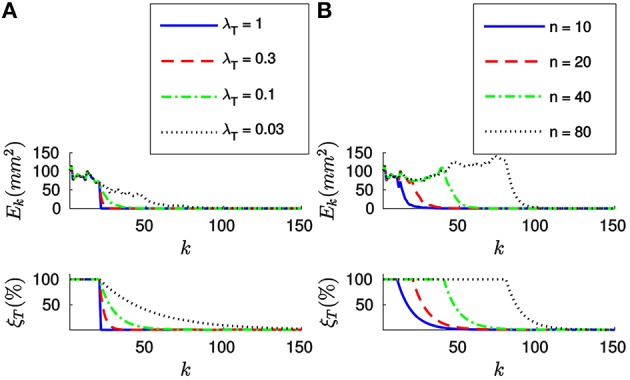
**(A)** Evolution of the sum of the squared collinearity errors *E*_*k*_ and the relative translation error ξ_*T*_ with the number of iterations for four different values of λ_*T*_, when applying the *full method* with *n* = 20. The bigger λ_*T*_, the faster the convergence. **(B)** Evolution of *E*_*k*_ and ξ_*T*_ for four values of *n* when λ_*T*_ = 0.1. We do not start updating the estimation until we have accumulated *n* events. After that point, the behavior of the system is very similar for every value of *n*.

Let us first apply the *full method* to the synthetic stream of events making *n* = 20. Figure 4A shows the evolution of both the sum of the squared collinearity errors *E*_*k*_ and the relative translation error ξ_*T*_ with the incoming events for four different values of λ_*T*_. We do not plot the relative rotation error ξ_*R*_, since it will always be zero in this case. Let us note that, for the first *n* events, we cumulate the information (updating *A*_*k*_, ***B***_*k*_ and **Γ**_*k*_) but we do not update the estimation of the pose. Consequently, the relative translation error remains stable. *E*_*k*_ is not stable because, in general, different points of the object will yield different collinearity errors. After *n* events we start updating the estimation, and we can see how both errors decay toward zero.

Comparing the results obtained with different values of λ_*T*_, we verify that the bigger λ_*T*_ the faster the convergence. As a matter of fact, we could consider making λ_*T*_ = 1. This value of λ_*T*_ makes the estimated translation equal to the optimal one for the last *n* events at every iteration. If we are confident enough in the accuracy of our tracking this constitutes an acceptable strategy.

Figure 4B shows the evolution of *E*_*k*_ and ξ_*T*_ for four different values of *n*, with λ_*T*_ = 0.1. We can see how the estimation is not updated until *n* events have elapsed, but once it does the behavior of the system is very similar for all values of *n*. This can be explained because the object is in this case static and the tracking is perfect. In real world scenarios, choosing *n* will require a tradeoff between acceptable velocities of the object—a smaller *n* results in a shorter reaction time—and stability in the presence of tracking errors—if *n* is big enough we can expect tracking errors to be canceled out. In order to illustrate this point, let us simulate some inaccuracy in the tracking and evaluate the algorithm again.

**Figure 5:**
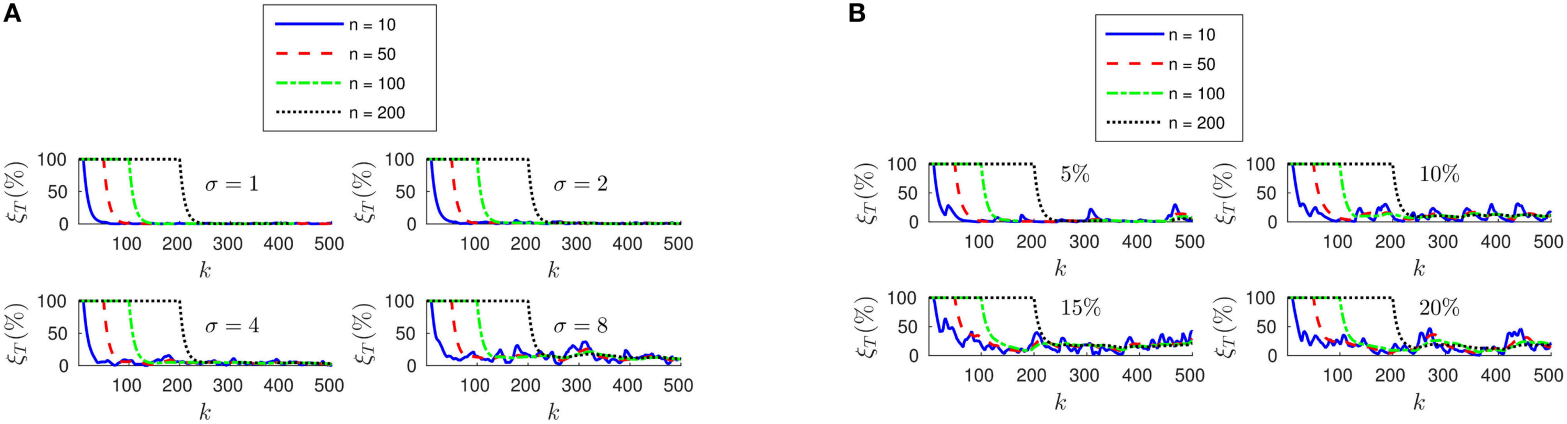
**(A)** Evolution of the relative translation error ξ_*T*_ when the tracking is not perfectly accurate. As the inaccuracy σ grows, the results of the pose estimation are strongly degraded. Additionally, when *n* is small the estimated pose has a greater variance. **(B)** Evolution of ξ_*T*_ with different percentages of wrongly assigned events.

To that end, we will assume that events are correctly assigned to the points generating them, but their position is noisy. This would correspond to a real case in which we are estimating the position of some markers, but there is some inaccuracy in this estimation. We will model the tracking errors as a gaussian noise with zero mean and standard deviation equal to σ. We plot in Figure 5A the evolution of the relative translation error ξ_*T*_ for four different values of σ (in pixels) and *n*. We verify that an inaccurate tracking strongly degrades the performance of our algorithm, resulting in increasing values for the final error. Additionally, for small values of *n* the estimated pose has a greater variance as the inaccuracy grows.

We next evaluate the effect of matching errors, i.e., when events are not correctly assigned to the point of the object generating them. Thus, we take a percentage of the events and randomly assign them to some other point of the object. We show in Figure 5B the evolution of the relative translation error ξ_*T*_ for four different percentages of wrongly assigned events. Again, we observe that matching errors degrade the performance of the algorithm.

These results allow us to conclude that the overall performance of the system is strongly dependent on the accuracy of the tracking. Additionally, we conclude that *n* should be chosen to be big enough to assure stability in the presence of tracking errors. The effect of this parameter will be more deeply analyzed in the next experiment, when treating real recordings. Let us note that increasing *n* will also result in a greater computation time.

**Figure 6:**
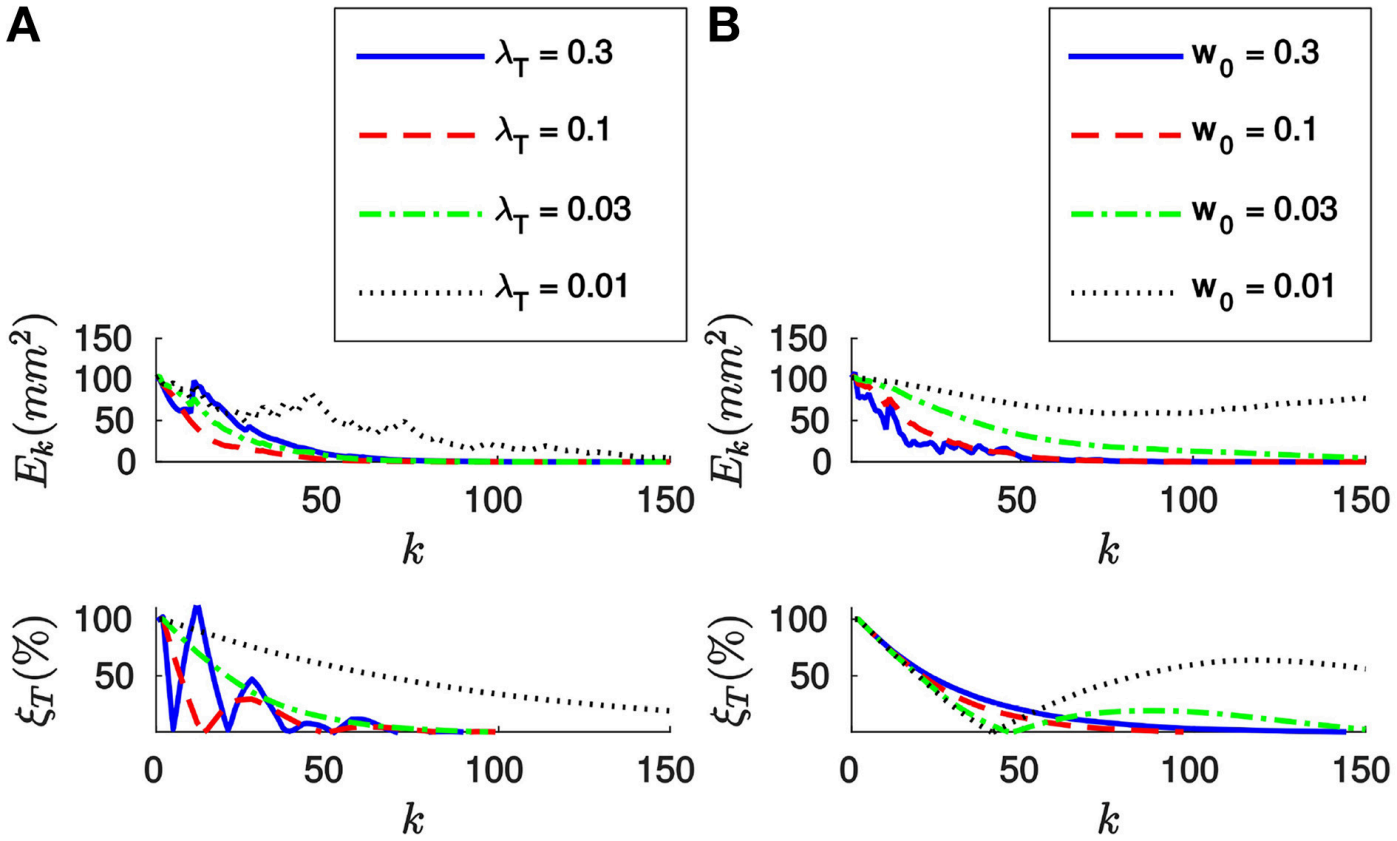
**(A)** Evolution of *E*_*k*_ and ξ_*T*_ with the number of iterations for four different values of λ_*T*_, with *w*_0_ = 0.1. The bigger λ_*T*_, the faster the convergence. However, too big a value of λ_*T*_ will cause the system to oscillate. **(B)** Evolution of *E*_*k*_ and ξ_*T*_ for four different values of *w*_0_, with λ_*T*_ = 0.03. Bigger values of *w*_0_ make the system more stable.

Next, let us estimate the translation by applying the *efficient method* to the synthetic stream of events. Figure 6A shows the evolution of both *E*_*k*_ and ξ_*T*_ for four different values of λ_*T*_, when *w*_0_ = 0.1. Let us note that when applying this strategy we do not set the value of *n*, and thus we will start updating the estimation from the first incoming event. As we can see in Figure 6A, both errors decay toward zero in this case as well, and the convergence is faster for bigger values of λ_*T*_. When applying the *efficient method*, however, big values of λ_*T*_ will cause the system to oscillate. Choosing λ_*T*_ will consequently require a tradeoff between speed of convergence and stability of the system.

Figure 6B shows the evolution of *E*_*k*_ and ξ_*T*_ for four different values of *w*_0_, when applying the *efficient method* with λ_*T*_ = 0.03. We observe that the system has a tendency to oscillate for small values of *w*_0_. This can be explained because small values of *w*_0_ result in a big “inertia” of the system. Let us remind the reader that we are iteratively updating the desired displacement Δ***T*** at each step. The parameter *w*_0_ controls how much we update Δ***T*** with every incoming event. Consequently, if *w*_0_ is too small, the desired displacement will continue to be big even when the collinearity errors are already small.

**Figure 7:**
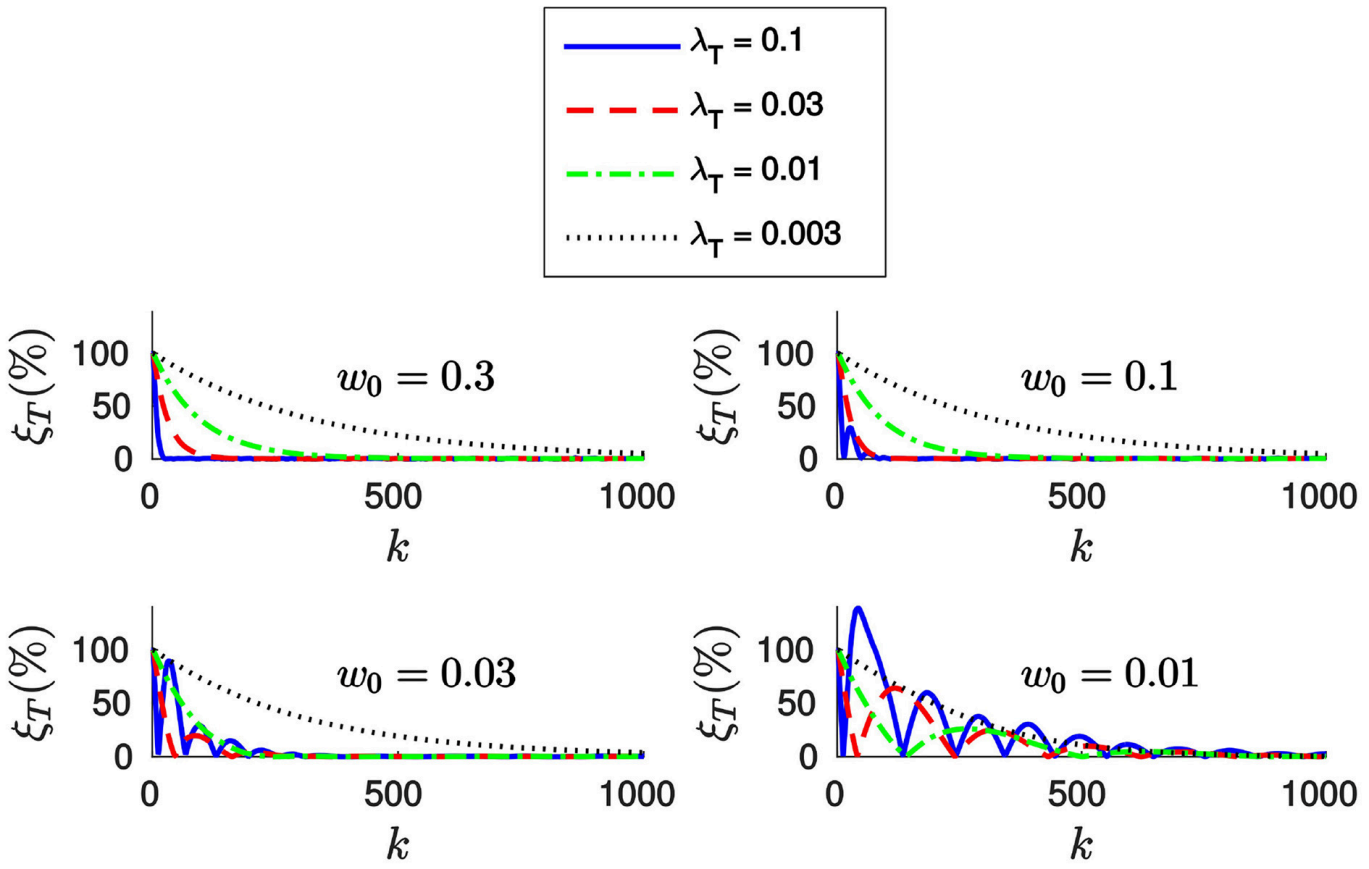
**Evolution of the relative translation error ξ_*T*_ with the incoming events for different values of *w*_0_ and λ_*T*_**. We verify that bigger values of *w*_0_ allow us to set a bigger λ_*T*_ without loosing stability. However, the system will become more sensible to tracking errors.

In order to further clarify this point, let us plot in Figure 7 the evolution of the relative translation error ξ_*T*_ for different values of *w*_0_ and λ_*T*_. As we can see, for small values of *w*_0_ the system tends to oscillate even when λ_*T*_ is small. This result suggests that one should assign big values to *w*_0_ (close to one) and to λ_*T*_, allowing to achieve fast convergence while the system remains stable. However, if the value of *w*_0_ is too big we will be assigning a great importance to the most recent events. This is actually equivalent to setting a small value for *n*, which will cause the system to be less stable in the presence of tracking errors.

We conclude that when applying the *efficient method w*_0_ and λ_*T*_ should be chosen together. We thus recommend values of *w*_0_ between 0.03 and 0.3, for λ_*T*_ between 0.01 and 0.1. In this case, the value of *w*_0_ has no effect on the computation time required.

#### 3.1.2. Rotation

We next test how the algorithm estimates only rotation. To that end, we make the initial estimation of the translation T^*^(0) equal to its true value ***T***, and set λ_*T*_ = 0. The initial estimation of the rotation is made $R_{o}^{*}\left(0\right)=I_{3}$, the 3 × 3 identity matrix.

The maximum distance in the synthetic object is ρ_*max*_ = 19.95 mm. Applying Equation (25) yields:

(32) $$\lambda_{r}^{opt}=0.0049{\text{N}}^{-1}{\text{mm}}^{-1}\approx \text{0}.\text{005 }{\text{N}}^{-1}{\text{mm}}^{-1}.$$

**Figure 8:**
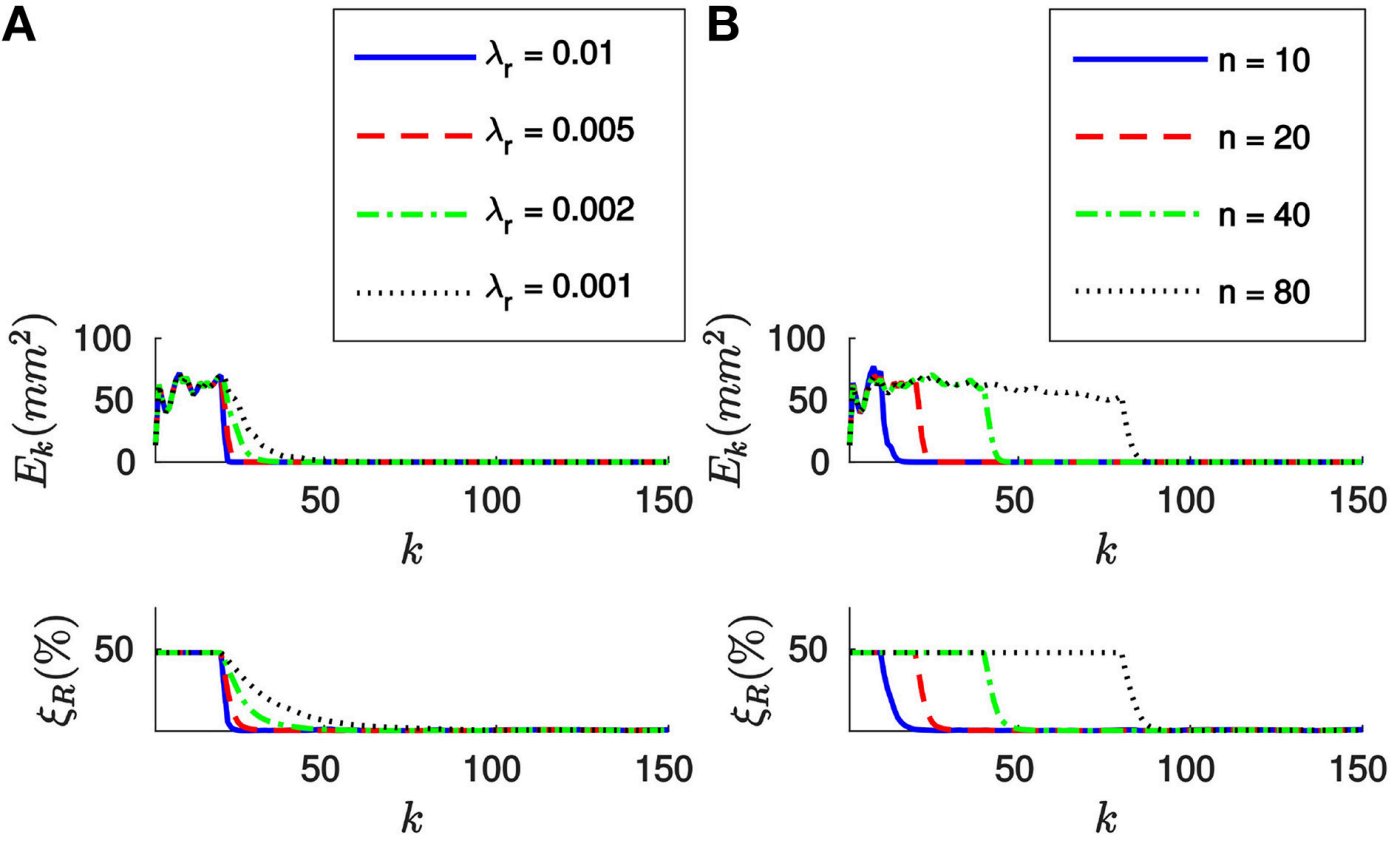
**(A)** Evolution of the errors for four different values of λ_*r*_ (in N^−1^mm^−1^), when *n* = 20. After *n* events both errors decay toward zero, the convergence being faster for bigger values of λ_*r*_. **(B)** Evolution of the errors for four different values of *n*, with λ_*r*_ = 0.005 (in N^-1^mm^-1^). After *n* events have elapsed, the behavior of the system is very similar for all values of *n*.

We will test different values of λ_*r*_ around $\lambda_{r}^{opt}$. Figure 8A shows the evolution of *E*_*k*_ and ξ_*R*_ with the incoming events for four different values of λ_*r*_, when applying the *full method* with *n* = 20. We verify that the results are very similar to the ones obtained in the case of the translation, with both errors decaying toward zero after *n* events. Analogously, the convergence is faster for bigger values of λ_*r*_. We verify that the system still yields stable results for $\lambda_{r}>\lambda_{r}^{opt}$. In this case, we experimentally determine that for values of λ_*r*_ greater than 0.01 the rotation fails to converge. We verify that Equation (25) provides good theoretical guidance for setting the order of magnitude of λ_*r*_. We thus recommend to simply set the value of λ_*r*_ as $\lambda_{r}^{opt}$.

Figure 8B shows the evolution of *E*_*k*_ and ξ_*R*_ with the incoming events for four different values of *n*, when $\lambda_{r}=\lambda_{r}^{opt}=0.005$ N^−1^mm^−1^. As in the case of the translation, after *n* events have elapsed both errors decay toward zero. Again, since the object is static and the tracking is perfect the behavior of the system is very similar for all values of *n*. For inaccurate tracking the same tradeoff applies as in the case of the translation, and we recommend values of *n* between 20 and 200.

**Figure 9:**
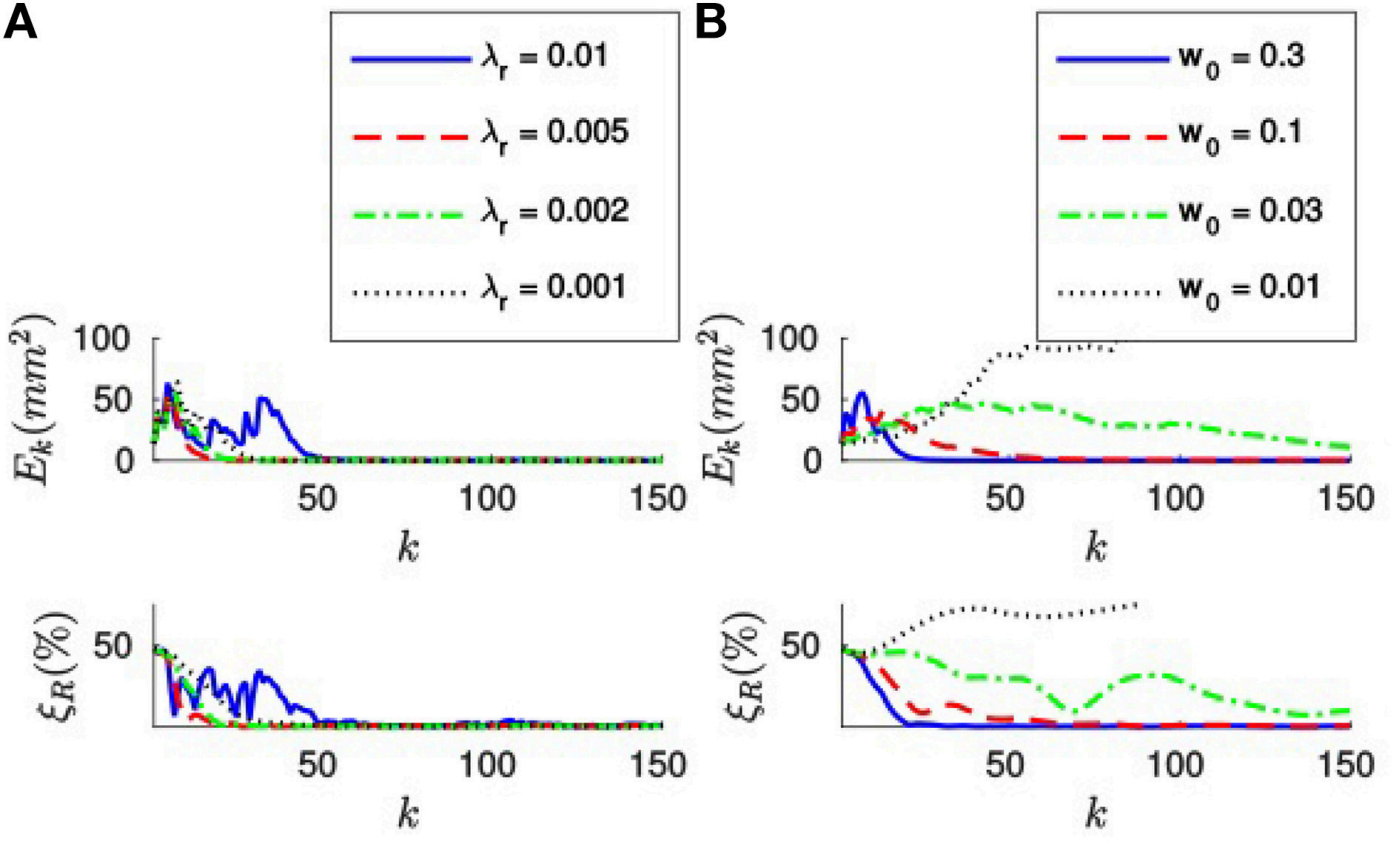
**(A)** Evolution of *E*_*k*_ and ξ_*R*_ for four different values of λ_*r*_, when *w*_0_ = 0.1. For big values of λ_*r*_ the system has a tendency to oscillate, and it might even fail to converge. **(B)** Evolution of the errors when λ_*r*_ = 0.001 N^−1^mm^−1^, for four different values of *w*_0_. When *w*_0_ is small, the system has a bigger tendency to oscillate.

Finally, let us apply the *efficient method* to estimate the rotation. Figure 9A shows the evolution of both *E*_*k*_ and ξ_*T*_ for four different values of λ_*r*_, when *w*_0_ = 0.1. We verify that when applying the *efficient method* the system has a bigger tendency to oscillate.

Figure 9B shows the evolution of the errors for four different values of *w*_0_, when λ_*r*_ = 0.002 N^-1^mm^-1^. As in the case of the translation, we verify that small values of *w*_0_ cause the system to oscillate. Analogously, too big a value of *w*_0_ will cause the system to be sensible to tracking errors. Consequently, we recommend the same fork of values for *w*_0_ between 0.03 and 0.3.

### 3.2. Real recordings

**Figure 10:**
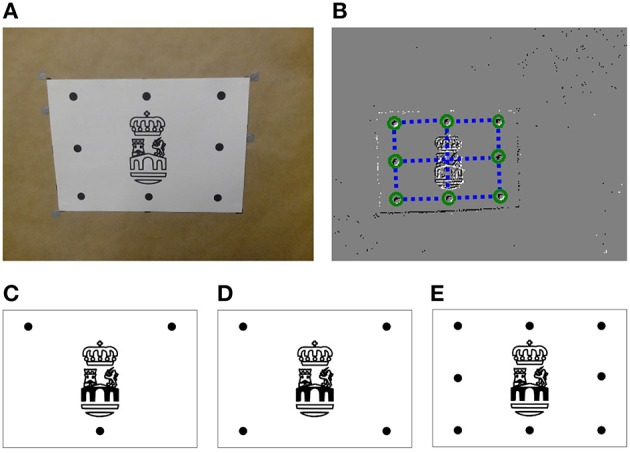
**(A)** Real object used in the experiments: black dots constitute the model of the object, while the logo in the center is used just for visual verification. **(B)** Output of the *Spring-Linked Tracker Set*: circles show the position of the simple trackers, while the dashed lines represent the springs linking them. **(C)** 3-point object **(D)** 4-point object **(E)** 8-point object.

Next, our algorithm is tested on real data obtained from an ATIS sensor, where an object moves and rotates in front the camera. As an object, we use a white piece of paper in which we printed some logo and a set of black dots (see Figure 10A). These dots constitute the model of the object.

In order to determine which point of the object has generated an event, we track these dots using the *Spring-Linked Tracker Set* presented in Reverter Valeiras et al. (2015). That work introduces a plane part-based tracking technique which describes an object as a set of simple trackers linked by springs. When the position of an incoming event is close enough to one of these simple trackers, then the event is assigned to that tracker, whose 2D location is updated. The springs then guarantee that the shape of the object keeps a certain coherence, while some variability—corresponding to the perspective transformation of the moving object—is allowed. When tracking a grid of points, the mean tracking errors reported in Reverter Valeiras et al. (2015) are below 3%, relative to the size of the object. This corresponds, for an object occupying a third of the camera's field of view, to an absolute tracking error of 3 px. From the results shown in Figure 5 we consider this value to be acceptable.

Figure 10B shows the output of the *Spring-Linked Tracker Set* when it is applied to one of our recordings. In the image, circles represent the position of each one of the simple trackers, while dashed lines depict the springs linking them. As we can see, we associate a simple tracker with each one of the black dots. Thus, when an event is assigned to one of these trackers, we consider that the event has been generated by the corresponding point of the object. In order to increase the accuracy of the method, we make the location of the event equal to the current position of the tracker, and then feed the resulting stream of clustered events to our P*n*P algorithm.

Ground truth values for numerical evaluation are obtained from an OptiTrack^1^system. OptiTrack is a motion capture system that outputs reliable values for the 3D pose of rigid bodies, provided that they are equipped with a number of infrared markers. Fixing markers on the object and the camera allows us to obtain their poses in the 3D space, from which we retrieve the pose of the object relative to the camera. Comparing this value with the estimation of our algorithm we compute the relative translation and rotation errors. Accuracy is characterized by the mean value of these errors computed for a whole recording.

We consider three different objects: the ones composed by three, four or eight points (see Figures 10C–E, respectively). This will allow us to evaluate the effect of the number of points on the accuracy of the algorithm. For all three objects the maximum distance is ρ_*max*_ = 136.01 mm. Applying Equation (25) yields $\lambda_{r}^{opt}\approx 0.0001$ N^−1^mm^−1^.

We make three different recordings for each one of the objects, producing a total of nine recordings. We identify them by their index, going from one to nine, where recordings #01 to #03 correspond to scenes containing the 3-point object. Recordings #04 to #06 contain the 4-point object, and #07 to #09 the 8-point object. In all of them the corresponding object is displaced and rotated in every direction. All recordings are cropped to have the same duration of 25 s and solved using the same set of parameters. The initial estimation of the pose is always made T^*^ = (0, 0, 0)^*T*^, $R^{*}\left(0\right)=I_{3}$.

We test the accuracy of both the *full method* and the *efficient method* on these real data. Additionally, we implement Lu's method (Lu et al., 2000) and apply it to our recordings as well. This allows us to evaluate our approach against a state of the art P*n*P algorithm.

**Figure 11:**
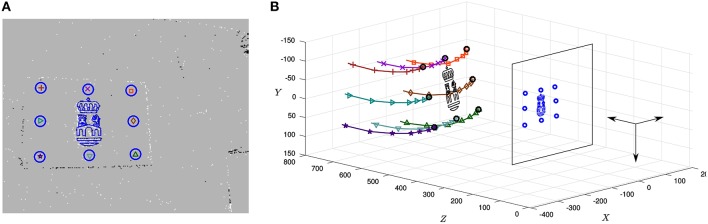
**(A)** Characteristic output of the P*n*P algorithm, corresponding to recording #09. The background of the image shows a snapshot of the ATIS recording, while events assigned to different points of the object are represented by different shapes (crosses, triangles, etc.). Circles indicate the reprojection of the object at its estimated pose: as we can see, the reprojection matches the corresponding events, showing that the algorithm is yielding good results on the focal plane. We reproject the logo as well, that matches the corresponding events. **(B)** 3D representation of the same instant, where the recent trajectories of the points of the object have been plotted using the same set of symbols.

Let us first apply the *full method* to the 9 recordings. Parameters are selected in the range giving stable results in the previous experiment. After several trials, they are experimentally set to *n* = 50, λ_*T*_ = 0.1 and λ_*r*_ = 0.0001 N^−1^mm^−1^. Figure 11A depicts the characteristic output of the P*n*P algorithm at a given instant, corresponding to recording #09. Here, the background of the image shows a snapshot of the ATIS output. Additionally, events assigned to different points of the object are indicated by different shapes (crosses, triangles, and so on), while circles represent the reprojection of the object at the pose estimated by the algorithm. We can see that, in general, circles surround the events generated by the corresponding points, showing that our method is yielding good results on the focal plane. We also reproject the logo, that as we can see matches the corresponding events. Figure 11B shows the state of the system at the same instant represented in the 3D space, where the camera's optical center has been placed at the origin. Recent trajectories of the points of the object have been plotted too, represented with the same set of symbols as in the 2D image. We show in Video 1 the output of the algorithm for this recordings: to the left we show results obtained on the focal plane, while 3D results are shown on the right side.

**Figure 12:**
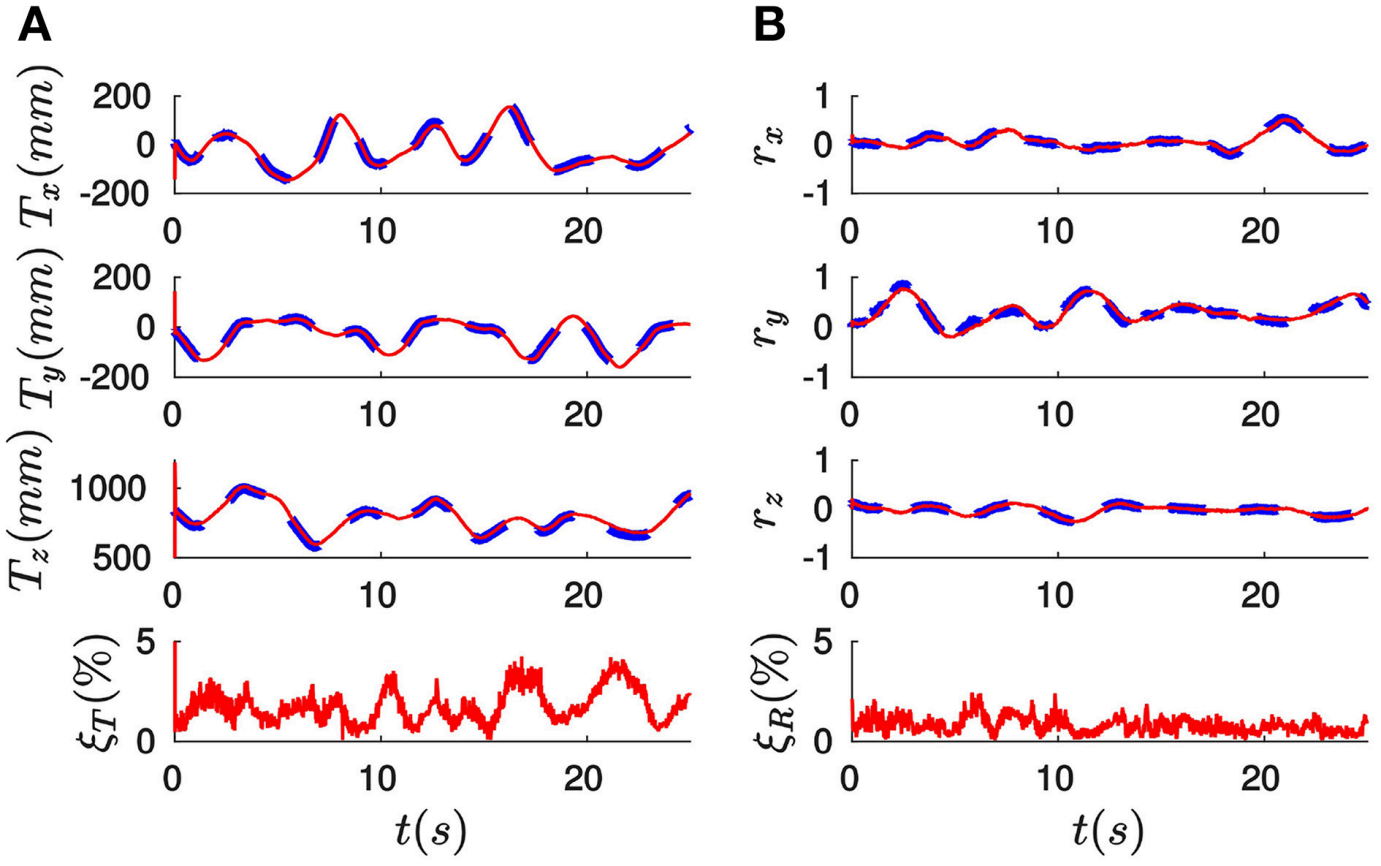
**(A)** Evolution of the three components of the translation vector ***T*** (in mm) for recording #09. Ground truth values are indicated by dashed lines, while the results produced by the *full method* are represented by solid lines. We verify that these two curves are coincidental. The resulting value of the relative translation error ξ_*T*_ is shown at the bottom: after a short initial transient its value remains always below 5%, yielding a mean value for the whole recording of just 1.79%. **(B)** Evolution of the rotation vector ***r***: estimated values are coincidental with ground truth values. This results in a mean value for the relative rotation error of 0.79%.

In order to illustrate pose estimation results produced by the algorithm, let us plot in Figure 12A the evolution of the three components of the translation vector ***T*** (in mm) for recording #09. In the figure, ground truth values are represented by dashed lines, and estimated values by continuous lines. We verify that these curves are coincidental, showing that the algorithm is correctly estimating translation. The relative translation error ξ_*T*_ is shown at the bottom of the figure: as we can see, after a short initial transient its value stabilizes to be always lower than 5%. This results in a mean value for the whole recording of just 1.79%. We denote this mean error $\bar{\xi_{T}}$, and use it to characterize the accuracy of a given approach.

Analogously, Figure 12B shows the three components of the rotation vector ***r***. As in the case of the translation, estimated values are coincidental with the ground truth references provided by the OptiTrack system. Consequently, the relative rotation error ξ_*R*_ is always below 5%, resulting in a mean value (denoted $\bar{\xi_{R}}$) of only 0.79%. These results allow us to conclude that the *full method* is correctly estimating the pose of the object for this recording.

**Figure 13:**
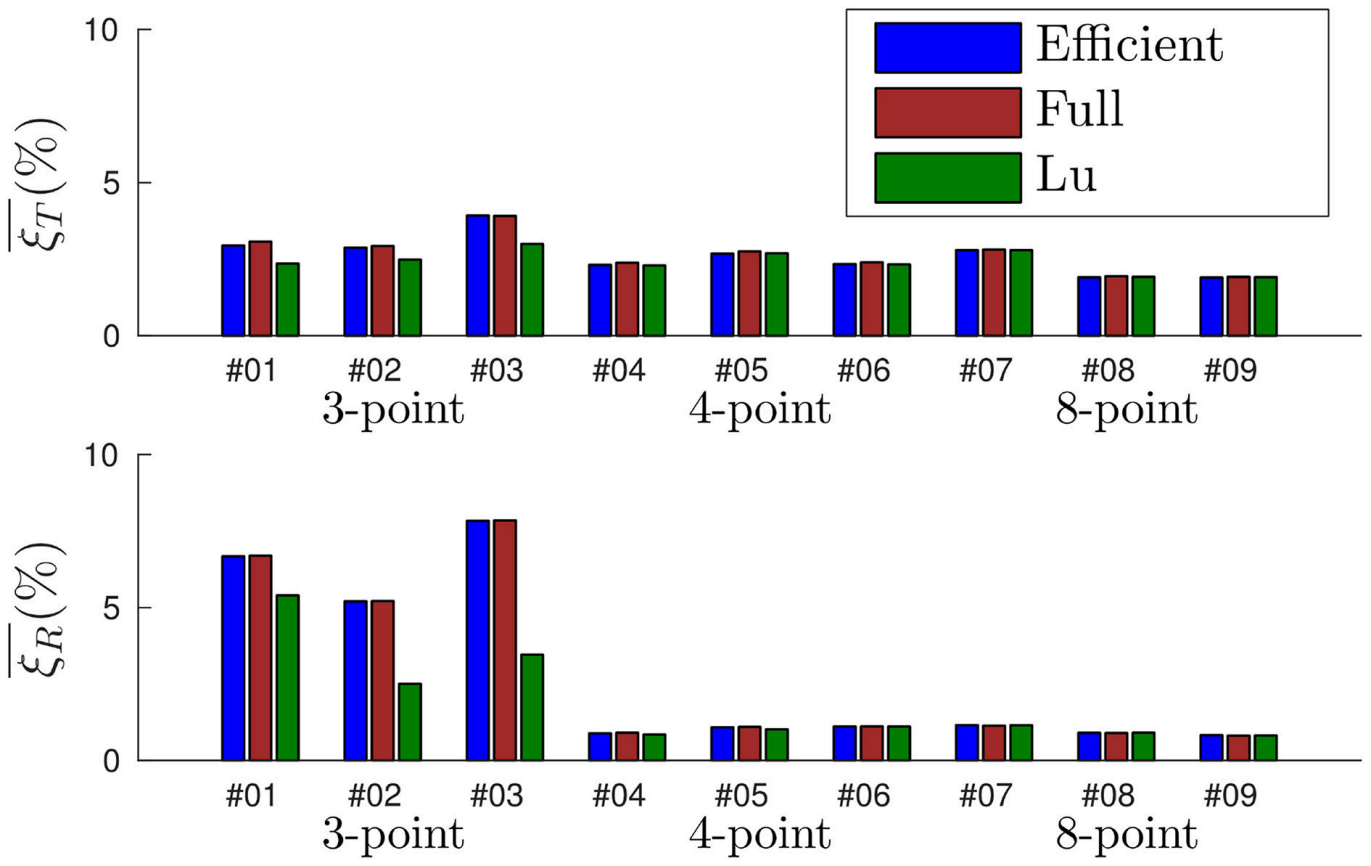
**Statistics of the errors obtained for each one of the nine recordings**. The *full method* and the *efficient method* yield equivalent results. When four or eight points are considered, the accuracy of our algorithm is equivalent to Lu's.

Let us next apply the *efficient method* to all the recordings with the following set of parameters: *w*_0_ = 0.1, λ_*T*_ = 0.1 and λ_*r*_ = 0.0001 N^−1^mm^−1^ (note that λ_*T*_ and λ_*r*_ take the same values as for the *full method*). Considering Lu's algorithm, the only parameter is *n*, that we experimentally set to *n* = 50. We show in Figure 13 the mean errors obtained for every recording with all three methods, where the relative translation error is shown on top and the relative rotation error at the bottom. Recordings of the same object are grouped together. From the results displayed in Figure 13 we can extract the following conclusions:

- The *full method* and the *efficient method* yield statistically equivalent results: for every recording, results obtained with both methods are almost identical. This proves the *efficient method* to be a valuable approximation.
- We cannot uniquely estimate the pose of an object with less than four points: when the 3-point object is considered, every method fails to produce accurate pose estimations. This is a known limitation of the P*n*P technique (Haralick et al., 1994), not specific to the event-based approach.
- When four or eight points are considered, pose is correctly estimated by our algorithm. Results obtained by our method are as reliable as the ones provided by Lu's, showing the accuracy of our approach. Both the *efficient method* and the *full method* produce errors in the same range of values, from 1.9% to 2.8% for the translation, and from 0.8 to 1.2% in the case of the rotation.
- Increasing the number of points to more than four does not improve the accuracy of the algorithm in the experiments. More than four points produce an overdetermined system, which is not always a guarantee of a better accuracy in the pose estimation. We hypothesize that, in this particular case, we have reached the limit of the algorithm because of the sensor's spatial resolution. A more thorough study with different stimuli, experimental conditions and tracking techniques is necessary to reach a firm conclusion.

In summary, when the object is composed by at least four points all three methods provide comparable accuracy. In order to establish a complete comparison between them we next evaluate their computation time.

### 3.3. Computation time

The presented experiments were carried out using a conventional laptop equipped with an Intel Core i7 processor and running Debian Linux. The algorithm was implemented both in Matlab and C++. Only the computation time of the C++ implementation is discussed. The code is not parallelized and a single core was used.

**Figure 14:**
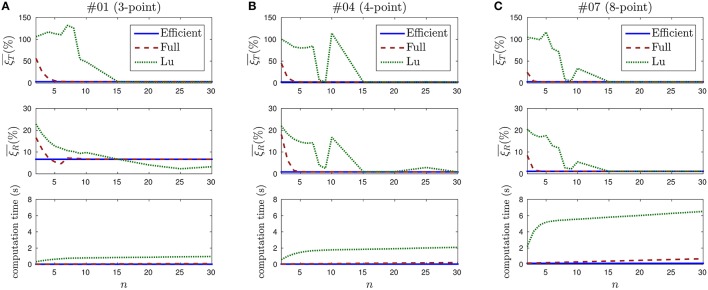
**Evolution of the pose estimation errors and the computation time with the value of *n***. **(A)** Results for recording #01. Top: mean translation error. Lu's method is unstable for small values of *n*, and its behavior is not predictable until *n* = 15. Middle: mean rotation error. Since this recording contains the 3-point object, rotation cannot be correctly estimated and rotation errors take big values for all three methods. Bottom: computation time. The *efficient method* is the fastest one, and it does not depend on *n*. **(B)** Results for recording #02. Rotation can be correctly estimated in this case. The computation time is greater than for recording #01, because more points in the object implies more events to treat. **(C)** Results for recording #07.

When applying the *full method* or Lu's method, the computation time depends on the value of *n* (the number of past events taken into account for updating the pose). We thus evaluate the evolution of both the computation time and the pose errors with the value of this parameter. We show in Figure 14 the results obtained for recordings #01, #04, and #07, where *n* takes values between 2 and 30. Only three recordings are shown for clarity reasons, results obtained for the remaining recordings are equivalent.

Figure 14A shows the results obtained for recording #01, which contains the 3-point object. On top, the evolution of the mean translation error $\bar{\xi_{T}}$ with the value of *n* is shown. If we analyze the *efficient method*, we observe that a straight line is obtained. This is an expected result, since this method does not depend on *n*. The value of the error is $\bar{\xi_{T}}=2.95\%$.

In the case of the *full method*, we observe that large errors are obtained for small values of *n*. This is also an expected result, as explained in Section 3.1. From *n* = 6 the error stabilizes at around 3%.

Finally, when applying Lu's method, we verify that the errors are even higher for small values of *n*, and they do not stabilize until *n* = 15. This can be explained because Lu's method is not incremental. Instead, it computes at each iteration the best solution for the last *n* events. Due to the nature of the neuromorphic camera, it often occurs that several consecutive events are generated by the same point of the object. Consequently, when Lu's method is applied with a small value of *n*, pose is often estimated using just one or two points of the object. This causes the estimation to be flawed, leading to large oscillations which result in the large observed errors. This is an indication that Lu's algorithm is not well-suited to the output of the neuromorphic camera.

The middle row of Figure 14A shows the mean rotation errors for recording #01. Since the 3-point object is considered in this case, rotation cannot be uniquely estimated. This results in large rotation errors for all three methods.

At the bottom of Figure 14A we show the computation time required for applying the P*n*P algorithm to recording #01. Values are averaged over 10 trials for each set of parameters. For the *efficient method* we observe a straight line at the value 0.017 s. In the case of the *full method*, we verify that the computation time grows linearly with *n*. If we analyze Lu's method, we verify that it also grows with *n*, linearly from *n* = 8.

We consider the computation time at *n* = 30 as a reference, since this value ensures stable solutions for all recordings with every method. The *efficient method* is then 6.1 times faster than the *full method* and 57.5 times faster than Lu's.

Figure 14B shows the results obtained for recording #04, that contains the 4-point object. Rotations can be correctly estimated in this case, the *efficient method* yields a mean rotation error $\bar{\xi_{R}}=0.89\%$. The *full method* and Lu's method stabilize around this value from *n* = 6 and *n* = 30, respectively. As observed before, small values of *n* cause the solution to oscillate, specially in the case of Lu's method.

At the bottom of Figure 14B the computation time required to solve recording #04 is shown. We verify that it follows the same previously observed pattern. However, the computation time is larger for every method, because considering more points implies more events to process. The computation time for the *efficient method* is 0.035 s. This is 6.0 times faster than the *full method* and 58.4 times faster than Lu's method (with *n* = 30).

Figure 14C shows the results for recording #07, that contains the 8-point object. Pose can be correctly estimated from *n* = 4 when applying the *full method*, and from *n* = 15 when Lu's method is chosen. When *n* = 30, the *efficient method* is 5.5 times faster than the *full method* and 52.4 times faster than Lu's.

It is important to emphasize that these results are implementation-dependent. Several optimization techniques can be applied, providing faster computation times. However, our implementation shows the *efficient method* to be around 50 times faster than Lu's method.

## 4. Discussion

An event-based P*n*P algorithm has been presented. To our knowledge, this is the first purely event-based solution to the P*n*P problem.

When computing the optimal translation of the object, we adapt a preexisting closed-form solution to our incremental approach. Rotation, however, cannot be solved in such a simple manner. Previous solutions employed complicated techniques to compute the optimal rotation for each frame, applying SVD to the solution of complex systems of equations. In our work, the rotation is estimated by simulating the evolution of a virtual mechanical system instead. This results in a simple yet robust solution to the problem, showing how event-based vision has the potential to simplify pose estimation tasks. We show how the high temporal resolution of incoming events makes it possible to apply simplifying assumptions without degrading the obtained results, but greatly reducing the number of operations per event. As an additional advantage, we consider our algorithm to be more intuitive and easier to implement than Lu's one. As a drawback, it requires the tuning of three parameters, while Lu's method has only one. However, we prove that there is a big range of values for which our method provides stable and accurate results.

When applying the *efficient method*, the resulting equations are very simple. Nevertheless, we prove that this approximation yields equivalent results to the *full method* when dealing with real recordings of moving objects. For the chosen set of parameters, when the object is composed of at least four points both the *efficient method* and the *full method* produce errors in the same range of values: from 1.9 to 2.8% for the translation, between 0.8 and 1.2% for the rotation. These values, very similar to the ones produced by Lu's algorithm, are sufficiently low to conclude that our method can correctly estimate 3D pose at a lower cost.

When the computation time of the different approaches is analyzed, we show that the *efficient method* is faster than the *full method*, and much faster than Lu's algorithm, while being equally accurate. For our precise implementation, the *efficient method* is around five times faster than the *full method* and 50 times faster than Lu's algorithm. Even if we are aware that these results are implementation-dependent, we consider the difference to be significant enough to conclude that the *efficient method* is faster, and thus recommend it as the standard choice. We claim that this gain in efficiency comes from the fact that our method is specifically adapted to handle the output of neuromorphic cameras.

As every P*n*P technique, our method requires matching 3D points with their 2D projections on the focal plane. The matching accuracy has consequently a strong impact on the overall performance of the system and the event-based P*n*P algorithm will benefit from any advances in event-based tracking or marker detection. For this reason, future research must put effort in the improvement of this layer. Future extensions of this research could also include the improvement of the mechanical model. Adding dashpots, for example, could allow for more aggressive constants, resulting in faster response times while avoiding oscillations. However, more complex models would likely increase computation loads, reducing its ability to satisfy real-time constraints: a compromise should be found with respect to this aspect.

Even taking into account reserves previously mentioned and according to the properties of the technique presented in this work, we have here the most advanced fully event-based P*n*P algorithm dedicated to silicon retinas. Since the estimated pose is updated with every event, the method takes full advantage of the high temporal resolution of the neuromorphic sensor. Moreover, the operations carried out with every event are reduced, guaranteeing real-time performance. Such property is highly demanded in embedded robotics tasks, such as visual servoing or autonomous navigation. This was the main motivation of this work, and provides the first stage to an entirely event-based formulation of visual servoing based on silicon retinas.

## Author contributions

DR formalized the theory and implemented the method. DR and SK designed and carried out the experiments. DR, SK, SI, and RB analyzed data. DR prepared figures and wrote the article. All authors reviewed the manuscript.

## Funding

This work received financial support from the LABEX LIFESENSES [ANR-10-LABX-65] which is managed by the French state funds (ANR) within the Investissements d'Avenir program [ANR-11-IDEX-0004-02]. This work received financial support from the EU Project [644096-ECOMODE].

### Conflict of interest statement

The authors declare that the research was conducted in the absence of any commercial or financial relationships that could be construed as a potential conflict of interest.

## Footnotes

^1^http://www.optitrack.com/.

## References

[B1] Boahen K. (2000). Point-to-point connectivity between neuromorphic chips using address events. IEEE Trans. Circuits Syst. II 47, 416–434. 10.1109/82.842110

[B2] Chong E. K. P. Zak S. H. (2001). An Introduction to Optimization. New York, NY: Wiley.

[B3] Clady X. Clercq C. Ieng S.-H. Houseini F. Randazzo M. Natale L.. (2014). Asynchronous visual event-based time-to-contact. Front. Neurosci. 8:9. 10.3389/fnins.2014.00009 24570652 PMC3916774

[B4] Coxeter H. S. M. (1961). Introduction to Geometry. New York, NY: Wiley.

[B5] Delbrück T. (1993). Silicon retina with correlation-based, velocity-tuned pixels. IEEE Trans. Neural Netw. 4, 529–541. 18267755 10.1109/72.217194

[B6] Delbrück T. Linares-Barranco B. Culurciello E. Posch C. (2010). Activity-driven, event-based vision sensors, in Proceedings of 2010 IEEE International Symposium on Circuits and Systems (ISCAS) (Paris: IEEE), 2426–2429.

[B7] Dementhon D. F. and Davis L. S. (1995). Model-based object pose in 25 lines of code. Int. J. Comput. Vis. 15, 123–141.

[B8] Etienne-Cummings R. Van der Spiegel J. Mueller P. (1997). A focal plane visual motion measurement sensor. IEEE Trans. Circuits Syst. I 44, 55–66.

[B9] Fischler M. A. Bolles R. C. (1981). Random sample consensus: a paradigm for model fitting with applications to image analysis and automated cartography. Commun. ACM 24, 381–395.

[B10] Haralick B. M. Lee C.-N. Ottenberg K. Nölle M. (1994). Review and analysis of solutions of the three point perspective pose estimation problem. Int. J. Comput. Vis. 13, 331–356.

[B11] Hartley R. Zisserman A. (2003). Multiple View Geometry in Computer Vision. Cambridge, UK: Cambridge University Press.

[B12] Huynh D. Q. (2009). Metrics for 3D rotations: comparison and analysis. J. Math. Imaging Vis. 35, 155–164. 10.1007/s10851-009-0161-2

[B13] Krammer J. Koch C. (1997). Pulse-based analog VLSI velocity sensors. IEEE Trans. Circuits Syst. II 44, 86–101.

[B14] Lagorce X. Meyer C. Ieng S.-H. Filliat D. Benosman R. (2014). Asynchronous event-based multikernel algorithm for high-speed visual features tracking. IEEE Trans. Neural Netw. Learn. Syst. 26, 1710–1720. 10.1109/TNNLS.2014.2352401 25248193

[B15] Lepetit V. Fua P. (2006). Keypoint recognition using randomized trees. IEEE Trans. Pattern Anal. Mach. Intell. 28, 1465–1479. 10.1109/TPAMI.2006.188 16929732

[B16] Lepetit V. Moreno-Noguer F. Fua P. (2008). EPnP: an accurate O(n) solution to the PnP problem. Int. J. Comput. Vis. 81, 155–166. 10.1007/s11263-008-0152-6

[B17] Lichtsteiner P. Posch C. Delbrück T. (2008). A 128 128 × 128 120 dB 15μs latency asynchronous temporal contrast vision sensor. IEEE J. Solid State Circuits 43, 566–576. 10.1109/JSSC.2007.914337

[B18] Lu C.-P. Hager G. Mjolsness E. (2000). Fast and globally convergent pose estimation from video images. IEEE Trans. Pattern Anal. Mach. Intell. 22, 610–622. 10.1109/34.862199

[B19] Mahowald M. (1992). VLSI Analogs of Neuronal Visual Processing: A Synthesis of Form and Function. PhD thesis, California Institute of Technology.

[B20] Montenegro Campos M. de Souza Coelho L. (1999). Autonomous dirigible navigation using visual tracking and pose estimation, in Proceedings of 1999 IEEE International Conference on Robotics and Automation Vol. 4 (Detroit, MI: IEEE), 2584–2589.

[B21] Murray R. M. Li Z. Sastry S. S. (1994). A Mathematical Introduction to Robotic Manipulation. Boca Raton, FL: CRC Press.

[B22] Ni Z. Ieng S. Posch C. Regnier S. Benosman R. (2014). Visual tracking using neuromorphic asynchronous event-based cameras. Neural Comput. 27, 925–953. 10.1162/NECO_a_00720 25710087

[B23] Posch C. Matolin D. Wohlgenannt R. (2008). An asynchronous time-based image sensor, in Proceedings of the IEEE International Symposium on Circuits and Systems (Seattle, WA: IEEE), 2130–2133.

[B24] Posch C. Matolin D. Wohlgenannt R. (2011). A QVGA 143 dB dynamic range frame-free PWM image sensor with lossless pixel-level video compression and time-domain CDS. IEEE J. Solid State Circuits 46, 259–275. 10.1109/JSSC.2010.2085952

[B25] Posch C. Serrano-Gotarredona T. Linares-Barranco B. Delbruck T. (2014). Retinomorphic event-based vision sensors: bioinspired cameras with spiking output. Proc. IEEE 102, 1470–1484. 10.1109/JPROC.2014.2346153

[B26] Reverter Valeiras D. Lagorce X. Clady X. Bartolozzi C. Ieng S.-H. Benosman R. (2015). An asynchronous neuromorphic event-driven visual part-based shape tracking. IEEE Trans. Neural Netw. Learn. Syst. 26, 3045–3059. 10.1109/TNNLS.2015.2401834 25794399

[B27] Reverter Valeiras D. Orchard G. Ieng S. H. Benosman R. B. (2016). Neuromorphic event-based 3d pose estimation. Front. Neurosci. 9:522. 10.3389/fnins.2015.00522 26834547 PMC4722112

[B28] Schweighofer G. Pinz A. (2006). Robust pose estimation from a planar target. IEEE Trans. Pattern Anal. Mach. Intell. 28, 2024–2030. 10.1109/TPAMI.2006.252 17108375

[B29] Skrypnyk I. Lowe D. (2004). Scene modelling, recognition and tracking with invariant image features, in Third IEEE and ACM International Symposium on Mixed and Augmented Reality. ISMAR 2004 (Arlington, VA: IEEE), 110–119.

