# Supplementary material for: An Event-Based Solution to the Perspective-n-Point Problem
Source: Front Neurosci. 2016 May 18;10:208. doi: 10.3389/fnins.2016.00208 (PMC4870282; doi:10.3389/fnins.2016.00208)
Supplement: Supplementary file 2 [file Appendices.pdf]

APPENDICES

A ITERATIVE UPDATE

Let us next define the following exponentially decaying set of weights:

$$w_j = w_0(1 - w_0)^j \tag{1}$$

with $w_0 < 1$. Then it is true that:

$$\lim_{j \to \infty} w_j = 0 \tag{2a}$$

$$\lim_{k \to \infty} \sum_{j=0}^{k-1} w_j = 1. \tag{2b}$$

Since the weights decay towards zero with j, we can approximate A_k by taking into account every past event e_{k-j} with $j = 0, 1, \ldots, k-1$:

$$A_k = \sum_{j=0}^{n-1} w_j (I_3 - L_{k-j}) \approx \sum_{j=0}^{k-1} w_j (I_3 - L_{k-j}). \tag{3}$$

Developing this expression A_k becomes:

$$\begin{aligned} A_k &\approx w_0(I_3 - L_k) + \sum_{j=1}^{k-1} w_j (I_3 - L_{k-j}) \\ &= w_0(I_3 - L_k) + \sum_{j=0}^{k-2} w_{j+1} (I_3 - L_{k-(j+1)}) \\ &= w_0(I_3 - L_k) + (1 - w_0) \sum_{j=0}^{k-2} w_j (I_3 - L_{(k-1)-j}) \\ &= w_0(I_3 - L_k) + (1 - w_0) A_{k-1}. \end{aligned} \tag{4}$$

In the case of $\boldsymbol{B}_k$, it becomes:

$$\boldsymbol{B}_k \approx \sum_{j=0}^{n-1} w_j (L_{k-j} - I_3) \boldsymbol{V}^*_{i(k-j)}. \tag{5}$$

Here, $\boldsymbol{V}^*_{i(k-j)}$ is the current estimated pose of the corresponding point of the object, whose value is dependent on R^* and $\boldsymbol{T}^*$, which change with every incoming event. Consequently, we cannot proceed as in the case of A_k in order to find an iterative expression for B_k. Nevertheless, and thanks to the high temporal resolution of the neuromorphic camera, we can assume that the pose changes slightly with each event. Since weights defined in (1) decay towards zero with j, we can assume that we are only taking into account

some recent events for which the estimated pose is approximately constant. Under this assumptions we can approximate the value of B_k in an analogous manner, making:

$$\boldsymbol{B}_k \approx w_0(L_k - I_3)\boldsymbol{V}^*_{i(k)} + (1 - w_0)B_{k-1}. \tag{6}$$

B JUSTIFICATION OF THE ROTATION

When we apply a resulting torque $\boldsymbol{\Gamma}$ to a body, the Newton's Second Law for rotation states that Taylor (2005):

$$\boldsymbol{\Gamma} = \frac{d\boldsymbol{H}}{dt}, \tag{7}$$

where $\boldsymbol{H}$ is the angular momentum of the body that takes the value:

$$\boldsymbol{H} = J\boldsymbol{\omega}. \tag{8}$$

Here, $\boldsymbol{\omega}$ is the angular velocity and J is a 3×3 symmetric matrix known as the inertia tensor. The form of J depends on how the mass of a body is distributed, and it gives an idea of how hard it is to accelerate the object around each one of the axis. However, since we are not modeling the behavior of a real mechanical system, we can imagine the mass of our virtual system to be equally distributed in all directions. This is equivalent to imagining the object to be embedded in a uniform sphere. The inertia tensor of a sphere has the form:

$$J = \alpha I_3, \tag{9}$$

where α is a real value, equal in the case of the sphere to $2ml^2/5$, l being the radius of the sphere and m its mass.

After these considerations (7) becomes:

$$\boldsymbol{\Gamma} = \alpha\frac{d\boldsymbol{\omega}}{dt}, \tag{10}$$

which is equivalent to the Newton's Second Law for translational motion. If we integrate this equation for a small period of time Δt, during which we suppose $\boldsymbol{\Gamma}$ to remain constant, and assuming zero initial angular velocity, we obtain the following rotation $\boldsymbol{r}$:

$$\boldsymbol{r} = \frac{1}{2}\alpha^{-1}\boldsymbol{\Gamma}\Delta t^2 = \lambda_r\boldsymbol{\Gamma}, \tag{11}$$

where we make $\lambda_r = \dfrac{\Delta t^2}{2\alpha}$.

C MAXIMUM TORQUE AND OPTIMAL λ_R

Developing the expression for the torque associated with event e_{k-j}, we get:

$$\begin{aligned}\Big(&R^*\boldsymbol{V}_{i(k-j)} \times L_{k-j}R^*\boldsymbol{V}_{i(k-j)}\\ &+ R^*\boldsymbol{V}_{i(k-j)} \times (L_{k-j} - I_3)\boldsymbol{T}^*\Big),\end{aligned} \tag{12}$$

which is expressed as the addition of two vectors. The first one of them is equal to the cross product of a vector with its own projection. Taking into account that $\|R\boldsymbol{V}\| = \|\boldsymbol{V}\|$, its norm is thus bounded by the expression:

$$\|R^*\boldsymbol{V}_{i(k-j)} \times L_k R^*\boldsymbol{V}_{i(k-j)}\| \leq \frac{\|\boldsymbol{V}_{i(k-j)}\|^2}{2}, \tag{13}$$

where the maximum value is attained when $R^*\boldsymbol{V}_{i(k-j)}$ and the line of sight form an angle of $\pi/4$ radians.

Next, $\|(L_{k-j} - I_3)\boldsymbol{T}^*\|$ is equal to the distance between $\boldsymbol{T}^*$ and the corresponding line of sight. If we assume that the estimated translation is close to its true value $\boldsymbol{T}^* \approx \boldsymbol{T}$, then it follows that:

$$\|(L_{k-j} - I_3)\boldsymbol{T}^*\| \approx \|(L_{k-j} - I_3)\boldsymbol{T}\| \leq \|\boldsymbol{V}_{i(k-j)}\|, \tag{14}$$

which happens when $R\boldsymbol{V}_{i(k-j)}$ is perpendicular to the line of sight.

The maximum torque will therefore be produced when both vectors in (12) take their maximum value, that is to say when $R\boldsymbol{V}_{i(k-j)}$ is perpendicular to the line of sight, and $R^*\boldsymbol{V}_{i(k-j)}$ forms an angle of $\pi/4$ radians with the line of sight. Fig. 1 shows the state of the system in this case. From the geometry it follows that:

$$\|\boldsymbol{\tau}_{k-j}\| \leq \frac{1+\sqrt{2}}{2}\beta w_j \|\boldsymbol{V}_{i(k-j)}\|^2.$$

If we call ρ_{max} to the maximum norm $\rho_{max} = \max_i\{\|\boldsymbol{V}_i\|\}$, then the resulting torque is bounded by the expression:

$$\|\boldsymbol{\Gamma}_k\| \leq \frac{1+\sqrt{2}}{2}\beta W \rho_{max}^2, \tag{15}$$

which depends on the dimensions of the considered object.

From the geometry, it follows that the angle formed by $R^*\boldsymbol{V}_{i(k-j)}$ and $R\boldsymbol{V}_{i(k-j)}$ is in this case $3\pi/4$ radians. We will accept the optimal value of λ_r, that we denote λ_r^{opt}, to be the one that causes the angle of the rotation applied in this case to be equal to $3\pi/4$ radians. λ_r^{opt} is thus given by the expression:

$$\lambda_r^{opt} = \frac{3\pi}{2(1+\sqrt{2})}\frac{1}{\beta W \rho_{max}^2}. \tag{16}$$

Let us note that the maximum value of the torque is very unlikely to be produced. Even if the geometry matches the right one, different points of the object are likely to produce events, usually yielding lower values of the torque. Consequently, λ_r^{opt} is a conservative value, and we can have stable systems with λ_r greater than λ_r^{opt}. However, we consider this expression to be an useful tool to determine the order of magnitude for this parameter.

REFERENCES

Taylor, J. (2005). *Classical Mechanics* (Sausalito, CA, USA: University Science Books)

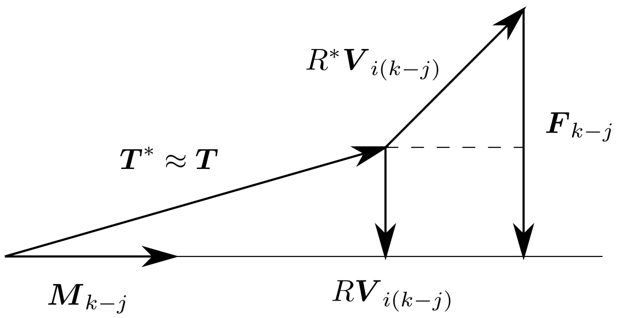

Figure 1. State of the system when the maximum torque is produced: $R^*\boldsymbol{V}_{i(k-j)}$ forms an angle of $\pi/4$ radians with the line of sight, causing $\|R^*\boldsymbol{V}_{i(k-j)} \times L_k R^*\boldsymbol{V}_{i(k-j)}\|$ to be maximum. At the same time, $R\boldsymbol{V}_{i(k-j)}$ is perpendicular to this same line of sight, making the distance between $\boldsymbol{T}$ and the line of sight maximum: $\|(L_{k-j} - I_3)\boldsymbol{T}\| = \|\boldsymbol{V}_{i(k-j)}\|$.
